# ID3 promotes erythroid differentiation and is repressed by a TAL1–PRMT6 complex

**DOI:** 10.1016/j.jbc.2024.108119

**Published:** 2024-12-22

**Authors:** Vivien Heller, Lei Wang, Edith Schneider, Mirjam Gerstner, Luana Bajer, Robin Decker, Halvard Boenig, Joern Lausen

**Affiliations:** 1 Department of Eukaryotic Genetics, Institute of Biomedical Genetics, University of Stuttgart, Stuttgart, Germany; 2 Institute for Transfusion Medicine and Immunohematology and German Red Cross Blood Service BaWüHe, Institute Frankfurt, Faculty of Medicine, Goethe University, Frankfurt, Germany

**Keywords:** erythropoiesis, basic helix–loop–helix transcription factor, epigenetics, histone methylation, cell differentiation, transcription regulation, transcription corepressor

## Abstract

Erythropoiesis is controlled by transcription factors that recruit epigenetic cofactors to establish and maintain erythrocyte-specific gene expression patterns while repressing alternative lineage commitment. The transcription factor TAL1 (T-cell acute lymphocytic leukemia 1) is critical for establishing erythroid gene expression. It acts as an activator or repressor of genes, depending on associated epigenetic cofactors. Understanding the epigenetic function of TAL1 during erythropoiesis is key to improving *in vitro* erythroid differentiation and understanding pathological erythropoiesis. Therefore, the regulatory mechanisms that control the function of TAL1 during erythropoiesis are under intense investigation. Here, we show that TAL1 interacts with protein–arginine–methyltransferase-6 (PRMT6) on the *ID3* (inhibitor-of-DNA-binding-3) gene in K562 and hCD34+ cells. The ID protein family is a critical transcriptional regulator of hematopoietic cell differentiation. We show that TAL1 and PRMT6 are present at the *ID3* promoter, and that TAL1 is involved in the recruitment of PRMT6. Here, PRMT6 epigenetically regulates ID3 expression by mediating dimethylation of histone 3 at arginine 2. Thus, TAL1–PRMT6 epigenetically represses ID3 expression in progenitors, which is relieved upon erythroid differentiation, leading to increased expression. Overexpression of ID3 in primary hCD34+ cells enhances erythropoiesis. Our results show that a TAL1–PRMT6 complex regulates genes important for erythropoiesis, such as *ID3*. Manipulation of ID3 expression may be a way to promote *in vitro* differentiation of hCD34+ cells into erythrocytes.

Hematopoietic stem cells give rise to progenitor cells, which can differentiate into the various blood cell lineages, such as erythrocytes. During erythropoiesis, transcription factors recruit transcriptional regulatory complexes, which can epigenetically modify chromatin by inducing activating or repressive histone modifications and DNA methylation. In this way, erythrocyte-specific gene expression patterns are established and maintained (1, 2, 3, 4, 5, 6). Knowledge of the gene regulatory mechanisms involved in this process is key to understanding erythroid differentiation and associated diseases. Furthermore, erythroid differentiation can be enhanced by experimentally altering the regulatory network that facilitates the erythroid gene expression program, for example in a therapeutic setting (7, 8, 9, 10, 11).

The transcription factor TAL1 (T-cell acute lymphocytic leukemia 1) is critical for erythropoiesis during embryogenesis (12, 13). TAL1 is one of the transcription factors required for fibroblast to erythroid transdifferentiation (14). Furthermore, TAL1 is involved in hematopoietic reprogramming of fibroblasts (15). TAL1 is expressed in megakaryocyte–erythroid progenitors (16, 17, 18, 19). During erythroid and megakaryocytic differentiation, TAL1 contributes to the activation of erythroid and megakaryocytic genes. However, TAL1 targets distinct gene sets in both lineages (20, 21, 22, 23, 24). We previously found that TAL1 is associated with the protein–arginine–methyltransferase-6 (PRMT6) on hematopoietic target genes (25, 26), but the specific role of this association is unclear. PRMT6 is able to asymmetrically dimethylate histone 3 at arginine 2 (H3R2me2a). This methylation is repressive because of its inhibition of trimethylation of lysine 4 on histone 3 (H3K4me3) (27, 28). PRMT6 is mostly associated with transcriptional repression but can also act as an activator (29, 30, 31). In concert with the transcription factor RUNX1, PRMT6 is able to maintain cell type–specific genes in a bivalent state (25, 26). We found that knockdown or inhibition of PRMT6 increases erythroid differentiation (32). Our novel observation that PRMT6 interacts with TAL1 highlights the role of TAL1 as a repressor of cell type–specific genes. The function of TAL1 as a transcriptional repressor is long known but less well understood than its activating role (33, 34, 35, 36). We hypothesize that a TAL1–PRMT6 complex takes part in the establishment of cell identity during the differentiation of progenitor cells into erythrocytes.

In particular, we found the TAL1–PRMT6 complex bound to the promoter of the *ID3* (inhibitor-of-DNA-binding-3) gene in K562 and hCD34 cells. ID3 belongs to a family of proteins, which contain a helix–loop–helix (HLH) domain but no DNA-binding basic domain. They interact with other HLH proteins and function as dominant negative inhibitors of basic-helix–loop–helix proteins (bHLH), in particular the ubiquitously expressed E-proteins, such as E47 (37). The four known ID genes (ID1, ID2, ID3, and ID4) have distinct expression patterns and functions in hematopoiesis (38, 39, 40, 41, 42, 43). ID3 may play a negative role in embryonic stem cell and induced pluripotent stem cell–derived hematopoiesis (39, 44). Furthermore, the combined ID1–ID3 deletion disturbs embryonic erythropoiesis (45). However, the role of ID3 in early hematopoiesis is not well understood.

In this study, we show that TAL1 and PRMT6 are present at the *ID3* promoter in K562 and hCD34 cells. Here, TAL1 is involved in the recruitment of PRMT6. PRMT6 epigenetically regulates ID3 expression by mediating H3R2me2a. Thus, TAL1–PRMT6 represses ID3 expression in progenitors, which is relieved upon erythroid differentiation, leading to increased ID3 expression. We further demonstrated that overexpression of ID3 enhanced erythropoiesis.

## Results

### TAL1 interacts with PRMT6

**Figure 1:**
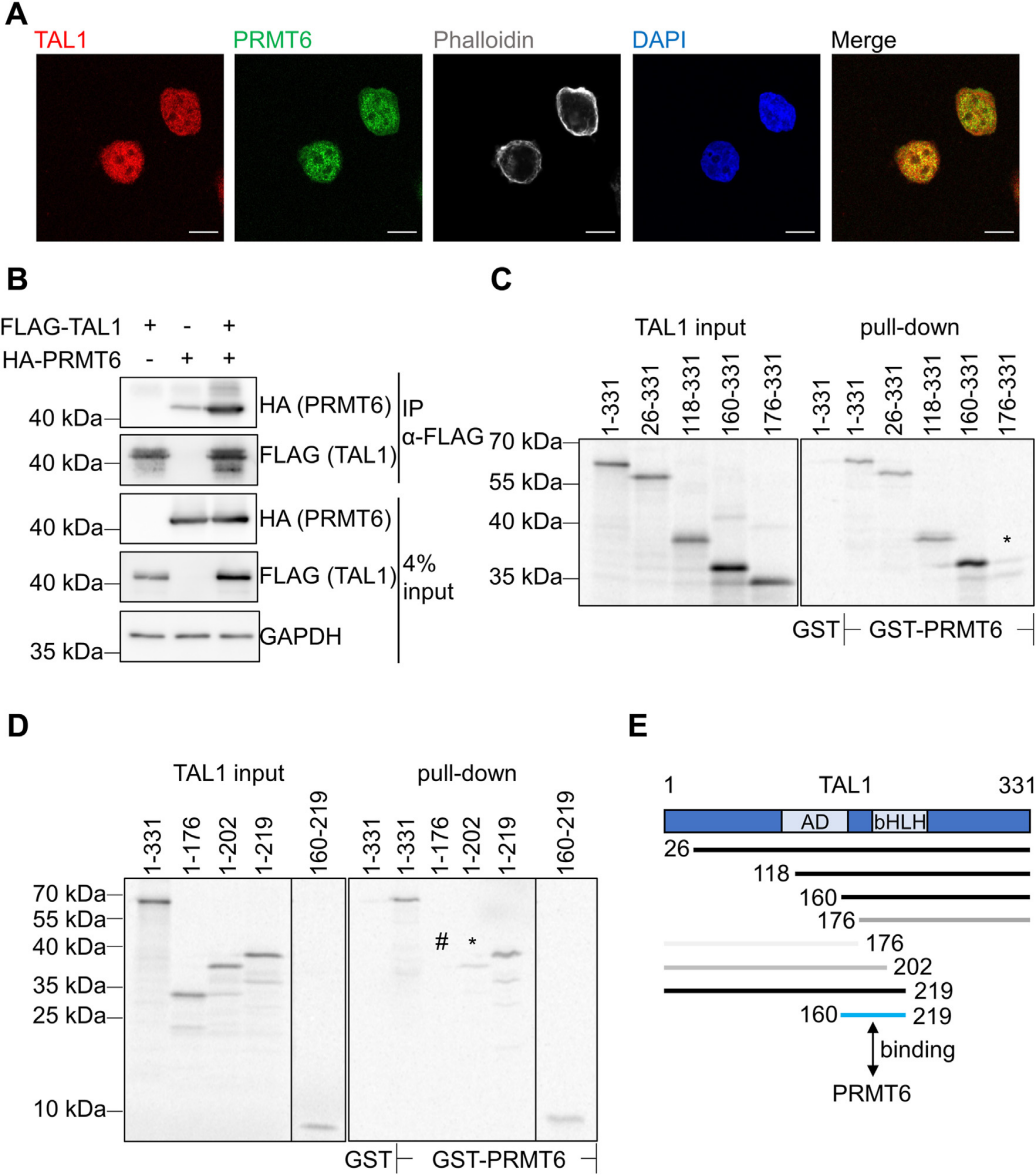
**TAL1 interacts with PRMT6.***A*, localization of TAL1 and PRMT6 in K562 cells with specific primary antibodies and AlexaFluor (488, 647)-labeled secondary antibodies. F-Actin-specific immunostainings plus nuclear counterstain (DAPI). Images are maximum intensity projections of several confocal sections. Scale bars represent 10 μm. *B*, coimmunoprecipitation of overexpressed HA-tagged PRMT6 and FLAG-tagged TAL1 in HEK293T cells. *C* and *D*, TAL1 and PRMT6 interact *in vitro*. GST pull-down with recombinant GST-PRMT6 and *in vitro* translated ^35^S-labeled full-length TAL1 (1–331) or smaller TAL1 fragments. *E*, schematic representation of TAL1 and PRMT6. Regions involved in interaction are marked as *black bars*, weak interaction marked as *gray bar*, *blue bar* represents amino acids 160 to 219 of TAL1 interacting with PRTM6. Numbers refer to amino acid (aa) numbers. AD, activation domain; bHLH, basic helix loop helix; DAPI, 4′,6-diamidino-2-phenylindole; GST, glutathione-*S*-transferase; HEK293T, human embryonic kidney 293T cell line; PRMT6, protein–arginine–methyltransferase-6; TAL1, T-cell acute lymphocytic leukemia 1.

Previous experiments indicated a relationship between TAL1, RUNX1, and PRMT6 on hematopoietic target genes (26, 32). Thus, we investigated whether TAL1 interacts with PRMT6. TAL1 and PRMT6 were found in the nucleus of K562 cells, and their location overlap (Fig. 1*A*). In addition, we demonstrated that TAL1 and PRMT6 associate by coimmunoprecipitation in transfected human embryonic kidney 293T (HEK293T) cells (Fig. 1*B*). Similarly, recombinantly produced full-length glutathione-*S*-transferase (GST)-PRMT6 interacted with *in vitro* translated ^35^S-labeled TAL1 in a GST pull-down assay (Fig. 1, *C* and *D*). Full-length TAL1 and smaller fragments of TAL1 interacted with PRMT6. Whereas the region of amino acid (aa) aa160–331 of TAL1 displayed strong interaction (Fig. 1*C*), a further 20aa deletion to aa176–331 resulted in loss of interaction. Fragments of TAL1 of aa1–176 as well as aa1–202 showed no or weak interaction in a GST pull-down (Fig. 1*D*). Based on these results, the interaction site of TAL1 with PRMT6 was narrowed down to aa160–219 of TAL1 (Fig. 1*E*). This region comprises of the DNA-binding region and the first helix. The interaction data support the notion that TAL1 and PRMT6 concomitantly bind to target genes.

### Common targets of TAL1 and PRMT6

**Figure 2:**
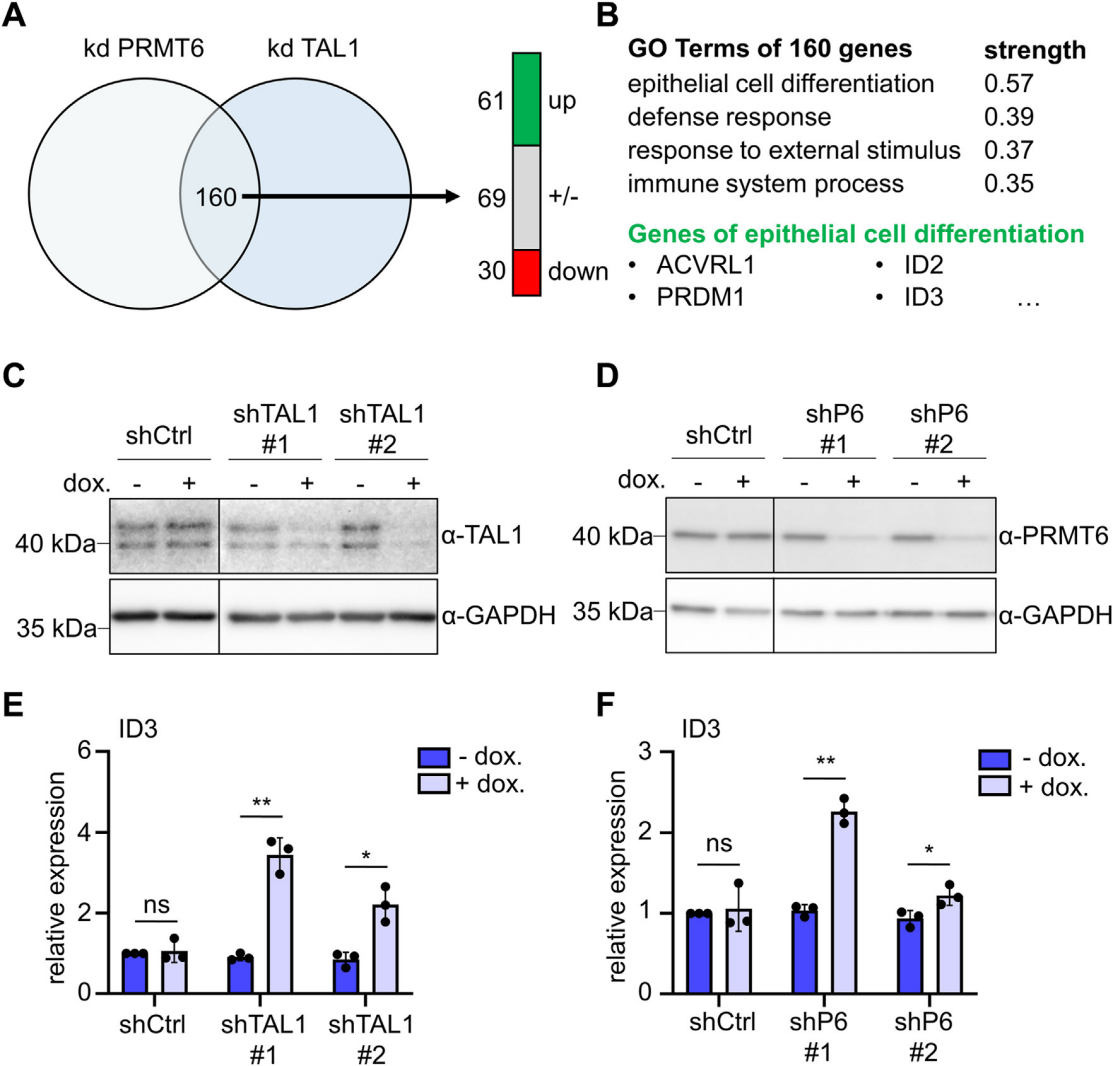
**TAL1 and PRMT6 have common target genes.***A*, analysis of gene expression after TAL1 or PRMT6 knockdown in K562 cells. Seven days upon knockdown, gene expression was determined and altered genes compared. About 160 genes are altered in both datasets. *B*, Gene Ontology (GO) term analysis of those 160 genes using the webtool “string” with default settings. Examples of annotated genes are given. *C* and *D*, stable inducible TAL1 (*C*) and PRMT6 (*D*) knockdown was established. Western blot shows knockdown of TAL1 (*C*) and PRMT6 (*D*) in K562 upon induction with 1 μM doxycycline for 7 days. GAPDH serves as loading control. shP6 = shPRMT6. *E* and *F*, ID3 expression was augmented upon TAL1 (*E*) or PRMT6 (*F*) knockdown. ID3 expression was determined by RT–quantitative PCR and normalized to GAPDH expression. Graphs show the means ± SD of four independent experiments (n = 4). *p* Values were calculated using Student's *t* test (∗*p* < 0.05; ∗∗*p* < 0.01). ID3, inhibitor-of-DNA-binding-3; ns, not significant; PRMT6, protein–arginine–methyltransferase-6; TAL1, T-cell acute lymphocytic leukemia 1.

To identify common target genes of TAL1 and PRMT6, we compared the genes altered by TAL1 and PRMT6 knockdown in K562 cells, respectively. Gene expression analysis (32) identified about 700 altered genes upon TAL1 knockdown, and expression of approximately 1000 genes was changed upon PRMT6 knockdown (Fig. 2*A*). We identified 160 genes that were simultaneously altered upon TAL1 and PRMT6 knockdown (Fig. 2, *A* and *B*). Gene Ontology term analysis of these 160 genes showed that genes annotated to have a function in epithelial cell differentiation were most significantly altered; this includes genes for receptors such as ACVRL1 and transcription factors such as PRDM1 (Figs. 2*B* and S4). In line with the observation that PRMT6 is mostly known as a repressor (25, 27, 28, 46), those genes were upregulated upon knockdown. In order to further examine a potential TAL1–PRMT6 corepressor complex, we established a doxycycline-inducible knockdown system for TAL1 (Figs. 2*C* and S5) and PRMT6 (Figs. 2*D* and S6). Our focus was on ID3 because it is known to be an inhibitor of bHLH proteins and plays, such as TAL1, a role in hematopoiesis (38, 39, 42). We demonstrated that knockdown of TAL1 and PRMT6 increased the expression of ID3 (Fig. 2, *E* and *F*). These results were confirmed by using a doxycycline-independent knockdown system for TAL1 and PRMT6 (Fig. S7, *A–F*). Taken together, these data indicate that TAL1 and PRMT6 act as repressors of ID3 expression.

### TAL1, E47, and PRMT6 bind to the *ID3* promoter in K562 cells

**Figure 3:**
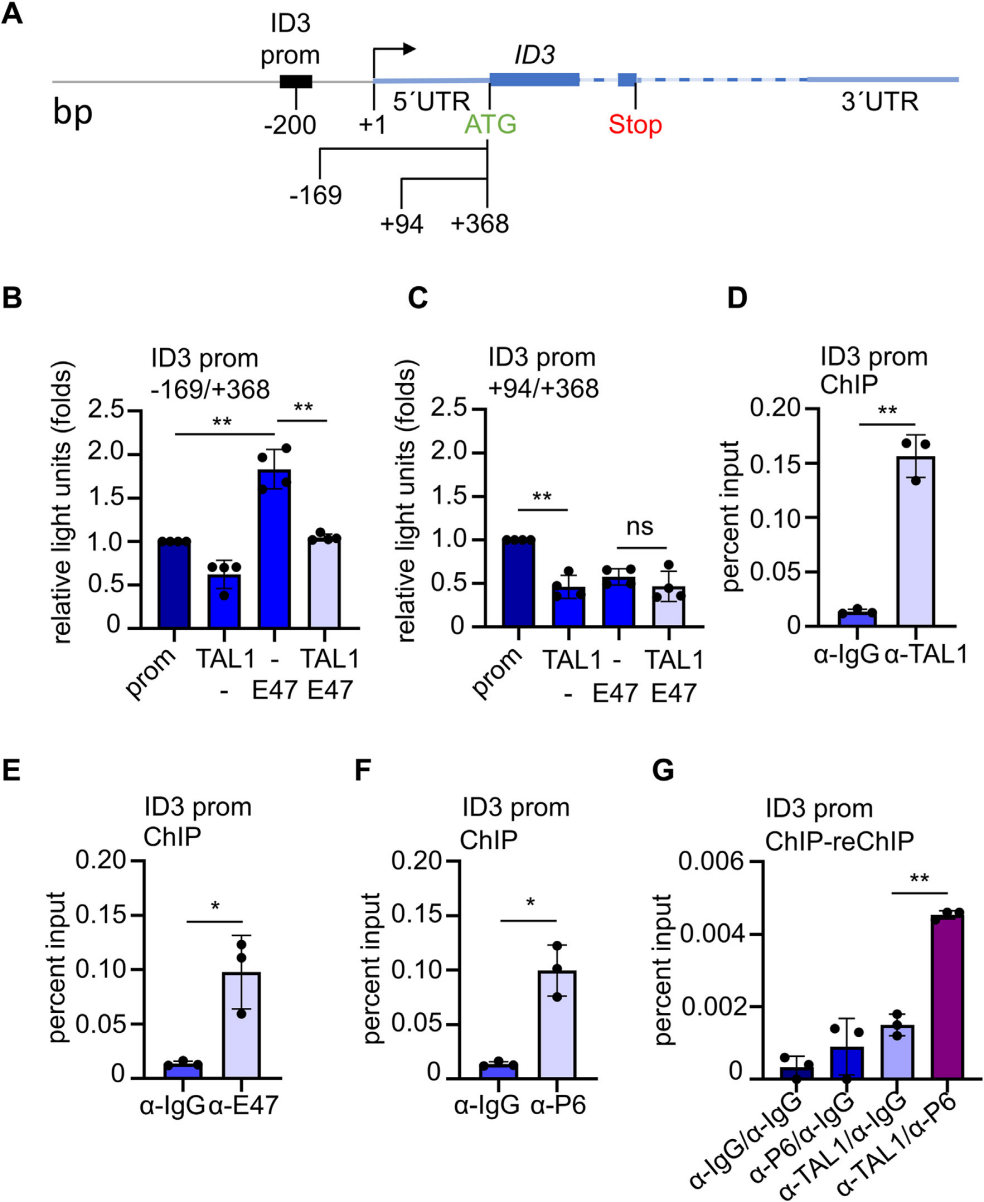
**TAL1, E47, and PRMT6 bind to the *ID3* promoter in K562 cells.***A*, schematic representation of the *ID3* locus. *ID3* promoter constructs are shown relative to the transcriptional start site. ChIP-Primer allow coverage of the proximal promoter area of ID3. *B* and *C*, TAL1 and E47 influence *ID3* promoter activity in a reporter gene assay. E47 activates the *ID3* promoter. TAL1 cotransfection decreased ID3 activity. Normalized values are given as relative light units, and the values gathered upon transfection of the promoter only are set as one. Graphs show the means ± SD of four independent experiments (n = 4). *D*–*F*, TAL1, E47, and PRMT6 bind to the *ID3* promoter in K562 cells. *G*, ChIP–reChIP analysis of TAL1 and PRMT6 in K562 cells. ChIP assays were quantified *via* quantitative PCR with specific oligos for the *ID3* promoter. The regions to which the oligos bind are shown relative to the transcriptional start site. Data are given as percent input. Graphs show the means ± SD of three independent experiments (n = 3). *p* Values were calculated using Student's *t* test (∗*p* < 0.05; ∗∗*p* < 0.01). ATG, start codon; bp, base pairs; +1 transcription start point; ChIP, chromatin immunoprecipitation; ID3, inhibitor-of-DNA-binding-3; PRMT6, protein–arginine–methyltransferase-6; Stop, stop codon; TAL1, T-cell acute lymphocytic leukemia 1.

The *ID3* gene is located on chromosome 1 with a length of 1865 base pairs (bp) and consists of two coding exons, a 5′UTR and a 3′UTR (Fig. 3*A*). The coding region has a length of 467 bp (47). In addition, ID3 interacts with E47 (37) (Fig. S8). Therefore, we investigated the influence of TAL1 and E47 on different *ID3* promoter reporter gene constructs in HEK293T cells (Fig. 3*A*). An *ID3* promoter reporter gene construct of −169/+368 bp was activated by E47, and this activation was decreased by coexpression of TAL1 (Fig. 3*B*). A shorter *ID3* promoter fragment from +94/+368 bp was not activated by E47, indicating that TAL1–E47 binding occurs close to the transcription start site (Fig. 3*C*). Subsequently, we performed a chromatin immunoprecipitation (ChIP) analysis with primer pairs close to the transcriptional start site and found that endogenous TAL1 and E47 bind to the *ID3* promoter in K562 cells (Fig. 3, *D* and *E*). K562 cells display erythroid properties but can be also differentiated toward the megakaryocytic lineage by 12-O-tetradecanoyl-phorbol-13-acetate (48). Thus, K562 cells can be used to study some aspects of TAL1 biology. We found that PRMT6 binds to the *ID3* promoter in K562 cells (Fig. 3*F*). Notably, PRMT6 binds to the *ID3* promoter together with TAL1 (Fig. 3*G*), as shown by ChIP–reChIP. TAL1, E47, and PRMT6 did not bind to a negative control region (Fig. S9). In conclusion, TAL1 and PRMT6 bind together to the *ID3* locus, and E47 also binds to the *ID3* locus.

### TAL1 recruits PRMT6 to the ID3 promoter

**Figure 4:**
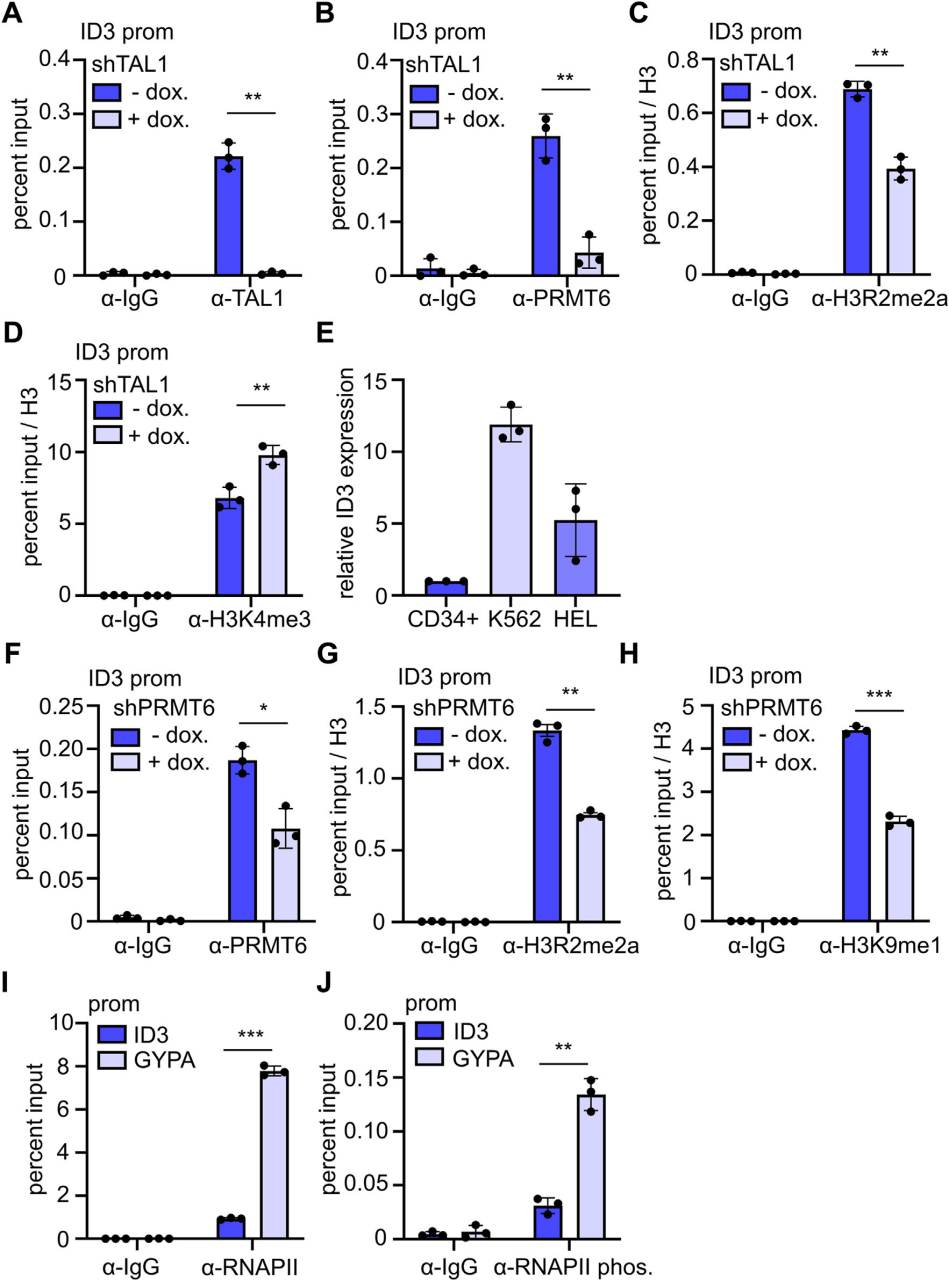
**TAL1 recruits PRMT6 to the *ID3* promoter in K562 cells.***A*, decreased enrichment of TAL1 at the *ID3* promoter in K562 cells upon TAL1 knockdown. *B*, decreased enrichment of PRMT6 at the *ID3* promoter after TAL1 knockdown. *C*, H3R2me2a was reduced after TAL1 knockdown. *D*, H3K4me3 was increased after TAL1 knockdown. ChIP assays were performed after TAL1 knockdown for 7 days. *E*, high expression of ID3 in K562 and HEL cells compared with hCD34+ cells. ID3 expression was determined by RT–quantitative PCR and normalized to GAPDH expression. Graphs show the means ± SD of three independent experiments (n = 3). Relative expression of hCD34+ cells is set as one. *F*, decreased enrichment of PRMT6 at the *ID3* promoter in K562 cells after a doxycycline-induced PRMT6 knockdown for 7 days. *G*, H3R2me2a was reduced after PRMT6 knockdown. *H*, H3K9me1 was reduced after PRMT6 knockdown. *I*, RNA polymerase II (RNAPII) was more enriched at glycophorin A (GYPA) promoter than at the *ID3* promoter in K562 cells. *J*, phosphorylated RNA polymerase II (RNAPII phos.) was more enriched at GYPA promoter than at the *ID3* promoter. Quantitative PCR values are shown as percent input. Values gathered for histone H3 modifications were normalized with a ChIP against unmodified histone H3. Graphs show the means ± SD of three independent experiments (n = 3). *p* Values were calculated using Student's *t* test (∗*p* < 0.05; ∗∗*p* < 0.01; and ∗∗∗*p* < 0.001). ChIP, chromatin immunoprecipitation; H3K4me3, trimethylation of lysine 4 on histone 3; H3R2me2a, dimethylation of histone 3 at arginine 2; ID3, inhibitor-of-DNA-binding-3; PRMT6, protein–arginine–methyltransferase-6; TAL1, T-cell acute lymphocytic leukemia 1.

To investigate TAL1 and PRMT6 binding to the *ID3* promoter more closely, we performed knockdown of TAL1 followed by a ChIP analysis in K562 cells. Knockdown of TAL1 reduced TAL1 binding to the *ID3* promoter as expected (Fig. 4*A*) and also reduced PRMT6 binding (Fig. 4*B*). The asymmetric H3R2me2a, which is triggered by PRMT6, was also reduced upon TAL1 knockdown (Fig. 4*C*). Conversely, H3K4me3 was enriched upon loss of TAL1 (Fig. 4*D*). However, H3K4me3 was already significantly detected in K562 cells. This is in line with the notion that there was a stronger expression of *ID3* in K562 cells than in hCD34+ cells (Fig. 4*E*). Subsequently, we knocked down PRMT6. This led to reduced PRMT6 occupancy at the *ID3* promoter (Fig. 4*F*). Although this reduction was only to 50%, H3R2me2a was reduced substantially (Fig. 4*G*). In addition, the repressive monomethylation of lysine 9 on histone 3 (H3K9me1) was reduced upon PRMT6 knockdown (Fig. 4*H*). We also observed that RNA polymerase II as well as phosphorylated RNA polymerase II was already present at the *ID3* promoter to some extent but not as much as at the *Glycophorin A* (*GYPA*) promoter in K562 cells (Fig. 4, *I* and *J*). We did not detect any enrichment for TAL1 or PRMT6 at the negative control region (Fig. S10). Our data strengthen the notion that TAL1 recruits PRMT6 to the *ID3* promoter, which acts repressively. Nevertheless, the *ID3* promoter exhibits both activating and repressive marks, yet the gene is expressed at a relatively low level.

### ID3 overexpression augmented erythroid differentiation

**Figure 5:**
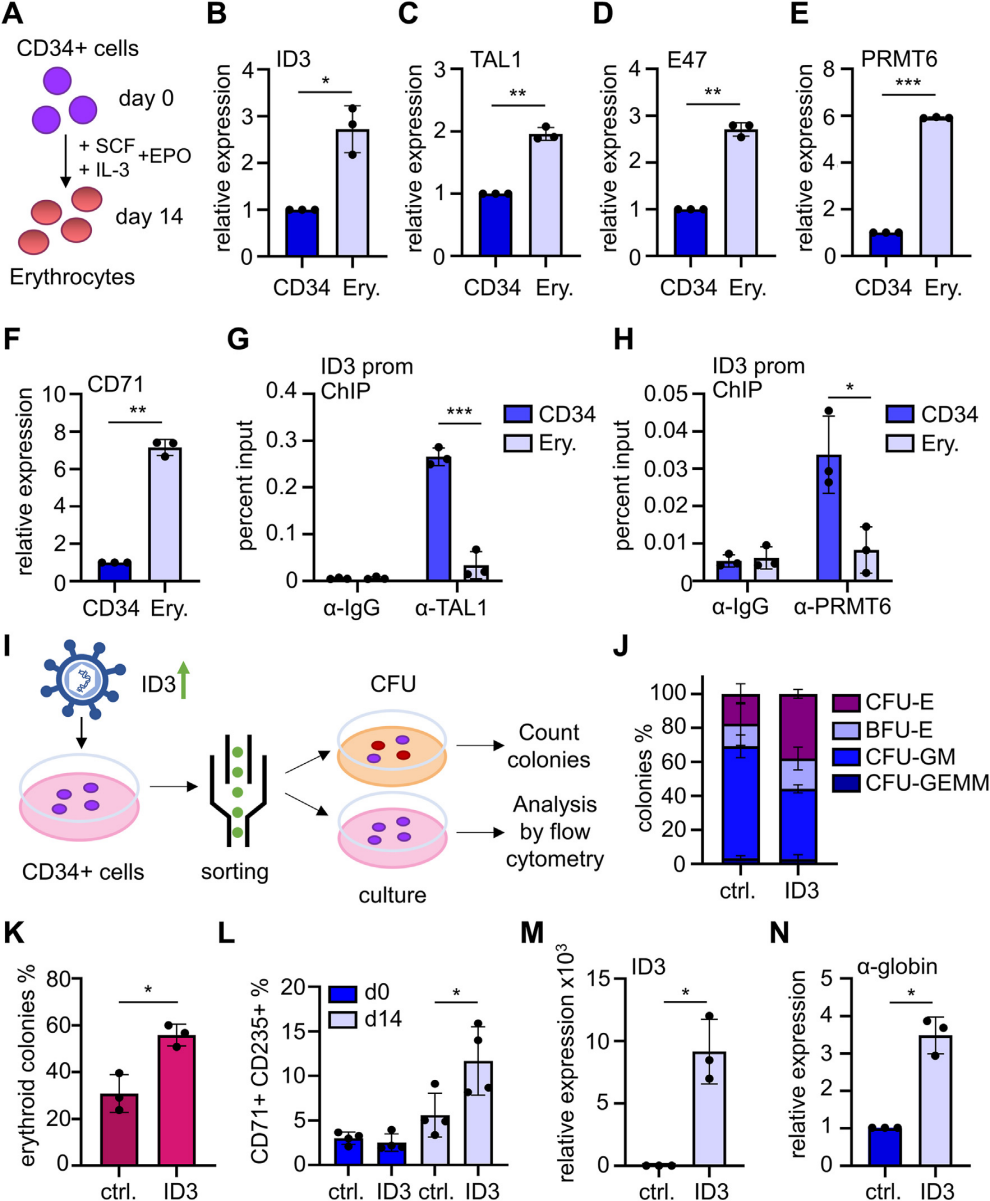
**ID3 overexpression augmented erythroid differentiation.***A*, primary human CD34+ cells were differentiated to erythrocytes. Expression of (*B*) ID3, (*C*) TAL1, (*D*) E47, (*E*) PRMT6, and (*F*) CD71 was measured on the mRNA level by RT–quantitative PCR. Expression was normalized to GAPDH expression, and the expression of undifferentiated hCD34+ cells was set as one. Graphs show the means ± SD of three independent experiments (n = 3). Decreased enrichment of (*G*) TAL1 and (*H*) PRMT6 at the *ID3* promoter in hCD34+ cells differentiated to erythroid cells compared with undifferentiated hCD34+ cells. ChIP assays were quantified *via* quantitative PCR with specific oligos for the *ID3* promoter. Graphs show the means ± SD of three independent experiments (n = 3). *p* Values were calculated using Student's *t* test (∗∗∗*p* < 0.001; ∗*p* < 0.05). *I*, scheme of the strategy to determine the influence of ID3 on differentiation. Transduced ID3-overexpressing cells were sorted for GFP-positive cells and subjected to colony-forming unit (CFU) assay or liquid culture (culture) in expansion medium for 14 days. As control (ctrl.), hCD34+ cells were transduced with an empty vector construct. *J*, ID3 overexpression led to an increase in erythroid colonies in CFU assay. *K*, representation of only erythroid colonies in CFU assay. Graphs show the means ± SD of three independent experiments (n = 3). *L*, overexpression of ID3 in hCD34+ cells led to increased number of CD71+ and CD235+ double-positive erythroid cells. Graphs show the means ± SD of four independent experiments (n = 4). *p* Values were calculated using Student's *t* test (∗*p* < 0.05). Expression of (*M*) ID3 and (*N*) α-globin in ID3-overexpressing hCD34+ cells. Expression levels were determined by RT–quantitative PCR and normalized to GAPDH expression. Graphs show the means ± SD of three independent experiments (n = 3). Relative expression of control hCD34+ cells (ctrl.) is set as one. *p* Values were calculated using Student's *t* test (∗*p* < 0.05). BFU-E, burst-forming unit-erythroid; CFU-E, colony-forming unit-erythroid; CFU-GEMM, common myeloid progenitor; CFU-GM, granulocyte-macrophage progenitor; ChIP, chromatin immunoprecipitation; ID3, inhibitor-of-DNA-binding-3.

Our data provide evidence for a regulation of ID3 expression by TAL1 and PRMT6. Thus, we examined if ID3 has an influence on erythropoiesis, which is TAL1 dependent (12, 13, 14). To further elucidate the role of ID3, we determined expression of ID3 upon differentiation of human CD34+ cells toward erythroid cells (Fig. 5*A*). ID3 is expressed at low levels in human CD34+ hematopoietic progenitors and is upregulated during erythroid differentiation (Fig. 5*B*). TAL1, E47, and PRMT6 also display an increased expression at the mRNA level during erythroid differentiation (Fig. 5, *C–E*). Erythroid differentiation was evaluated by mRNA expression of the erythroid marker genes CD71 (Fig. 5*F*) and CD235 (Fig. S11*A*) and verified by flow cytometry (Fig. S12). Taken together, our data indicate that increased ID3 expression is associated with erythroid differentiation. To analyze TAL1 binding to the *ID3* promoter, we performed ChIP analysis in undifferentiated hCD34+ cells and upon erythroid differentiation (Fig. 5*G*). TAL1 binding to the endogenous *ID3* promoter was decreased upon erythroid differentiation. In addition, we observed reduced PRMT6 binding at the *ID3* promoter upon erythroid differentiation of hCD34+ cells (Fig. 5*H*). To investigate the impact of ID3 expression on erythropoiesis, we overexpressed ID3 in hCD34+ cells. Subsequently, we performed a colony-formation unit assay and a differentiation analysis of ID3-overexpressing cells in liquid culture by flow cytometry (Fig. 5*I*). We observed promoted outgrowth of erythroid colonies upon ID3 overexpression (Fig. 5, *J* and *K* and S11*C*). ID3-overexpressing hCD34+ cells counted 25% more erythroid colonies than the control (Fig. 5*K*). In addition, ID3-overexpressing hCD34+ cells that were cultured in expansion media without induction of erythroid differentiation exhibited an increased number of CD235+ and CD71+ double-positive erythroid cells (Figs. 5*L* and S12). To analyze the impact of ID3 on gene expression associated with erythroid expression, we overexpressed ID3 in hCD34+ cells (Fig. 5*M*). This also resulted in an increase of α-globin expression in ID3-overexpressing hCD34+ cells (Fig. 5*N*).

Taken together, ID3 expression is increased upon erythroid differentiation and augments *in vitro* erythroid differentiation.

## Discussion

The transcription factor TAL1 is essential for erythroid gene expression (23, 49). Increased expression of TAL1 in human CD34+ cells augments erythropoiesis (50). We previously found that the transcription factors RUNX1 and TAL1 are associated with corepressor PRMT6 on a subset of erythroid genes in progenitors, and this association is lost upon erythroid differentiation (26). In line with the notion that PRMT6 represses erythroid genes, the knockdown of PRMT6 increased erythropoiesis (32). Here, we report that TAL1–PRMT6 repress the *ID3* gene in progenitor cells and that ID3 augments erythropoiesis.

### Association of TAL1–PRMT6

Our data show that the transcription factors RUNX1 and TAL1 are associated with the corepressor PRMT6 on some erythroid genes in hematopoietic progenitors. This association was lost upon erythroid differentiation (26). Knockdown of RUNX1 led to decreased PRMT6 binding, while some PRMT6 was still detectable upon loss of RUNX1 (25). Thus, we suspected that additional transcription factors contribute to PRMT6 recruitment. Here, we found that the interaction of TAL1 and PRMT6 takes place at the amino acids 160 to 219 of TAL1, which includes the basic domain and the first helix. It is conceivable that binding of PRMT6 in that region influences DNA binding or interaction with other cofactors (36, 51). Reduction of TAL1 leads to reduced PRMT6 binding (Fig. 4*B*). Thus, the central regulator of hematopoiesis TAL1 contributes to the recruitment of PRMT6 to target genes. Other such transcription factors are LEF1 (52), RUNX1 (25), Pparγ (53, 54), the estrogen receptor (55), and the androgen receptor (56). The observation that some of the PRMT6-recruiting transcription factors are found together at promoters hints toward the notion that they concomitantly recruit PRMT6.

### TAL1–PRMT6 regulates ID3

Knockdown of TAL1 led to alterations in about 700 genes, and the knockdown of PRMT6 altered approximately 1000 genes. About 160 genes were altered in both datasets. This relatively small number may indicate that the loss of PRMT6 or TAL1 at their target genes might not necessarily lead to immediate alteration in gene expression. This is the case for *GYPA* and *CD41*, which are verified target genes of TAL1 and PRMT6, but do not appear in our list of altered genes (25, 32, 57). Possibly, loss of TAL1 does not lead to reduced PRMT6 binding at all loci. In addition to that, loss of PRMT6 might not be sufficient to alter gene expression in all cases, because the underlying chromatin modification is stable and does not allow for timely gene expression alteration. The majority of altered TAL1 and PRMT6 target genes, which are concomitantly altered, are upregulated. This is in line with the notion that TAL1 and PRMT6 are associated within a corepressor complex. However, a subset of genes was downmodulated upon TAL1 or PRMT6 loss (Fig. 2*A*). In these cases, TAL1–PRMT6 might have activating roles, as PRMT6 can also act as an activator depending on the genetic context (29, 30, 31, 58).

The *ID3* promoter is bound by a TAL1–PRMT6 complex, as observed by ChIP–reChIP in K562 cells. However, TAL1 binding to this region was not detected in ChIP–Seq analysis of hematopoietic cells (59). E47, the heterodimerization partner of TAL1, can be detected at the *ID3* promoter, and TAL1 and E47 interplay at the promoter in a reporter gene setting. Notably, knockdown of TAL1 leads to loss of PRMT6 binding at the endogenous *ID3* promoter. H3R2me2a, which is mediated by PRMT6, is reduced upon loss of TAL1. Also, PRMT6 knockdown reduced H3R2me2a and the repressive H3K9me mark. These data are indicative of a setting in which TAL1 recruits PRMT6, and this leads to H3R2me2a. In line with the notion that H3R2me2a acts negatively on H3K4me3 (27, 28), this mark is increased upon TAL1 knockdown. However, the *ID3* promoter is not in a silenced state, as H3K4me3 can be detected to some degree at the promoter. This, and the finding that the *ID3* promoter is less active than the GYPA promoter in a ChIP with RNA polymerase II, seems to indicate that the *ID3* promoter is in a bivalent state.

### ID3 influences TAL1 activity and augments erythropoiesis

Inhibitor of DNA-binding proteins, such as ID3, are able to interact with bHLH transcription factor of the E-protein family, such as E47 and repress their function (37). The family of ID proteins have a profound impact on hematopoiesis (38, 45). In particular, combined loss of Id1 and Id3 in mice leads to multiple defects in erythropoietic differentiation including a downregulation of β-globin (45). This observation is consistent with our finding that ID3 expression is upregulated during erythroid differentiation (Fig. 5). Although TAL1 expression was elevated in erythroid cells (Fig. 5*C*), TAL1 binding was reduced upon erythroid differentiation (Fig. 5*G*). This occurs alongside with the reduction of PRMT6 binding. Thus, TAL1 and PRMT6 suppress ID3 in undifferentiated hCD34+ cells, and this repression is released upon erythroid differentiation when TAL1 and PRMT6 depart from the *ID3* promoter. The differential binding of TAL1 to target genes during differentiation is an important subject of future examinations. To further examine the function of ID3 in erythropoiesis, we overexpressed ID3 in primary human CD34+ cells, which led to an increase in erythroid differentiation (Fig. 5). Our data show for the first time that ID3 expression can lead to increased erythroid differentiation of primary human CD34+ cells. TAL1 and E47 bind as a heterodimer to the *ID3* promoter. TAL1 recruits PRMT6, leading to repressed ID3. The notion that ID3 can inhibit E47 (42) suggests a role of ID3 in the regulation of TAL1 heterodimerization with E47. This aspect needs further investigation. Taken together, our findings open the possibility to enhance differentiation of progenitor cells to erythrocytes by altering ID3 expression.

## Experimental procedures

### Cell culture

HEK293T cells (DSMZ no. ACC 635), HEL (DSMZ no. ACC 11), and K562 (DSMZ no. ACC 10) were cultured as previously described (52). Knockdown of TAL1 and PRMT6 mediated by shRNA was accomplished using pInducer10 vectors (60). Sequences of shRNA are provided (Table S1). After 7 days of sustained TAL1 or PRMT6 knockdown, the experiments were performed. Generation and production of lentiviral virus as well as transduction was performed as described previously (52). Pseudonymized primary human CD34+ cells (collected with written and informed consent of the donors in accordance with the Declaration of Helsinki and approval of the Ethics Committee 329/10, German Red Cross Blood-Donation-Center, Frankfurt) were expanded in StemSpan SFEM II (STEMCELL Technologies) supplied with StemSpan CD34+ Expansion Supplement (STEMCELL Technologies). To differentiate hCD34+ cells, the media were supplemented with Erythroid Expansion Supplement (STEMCELL Technologies) for 14 days. For expression analysis upon ID3 overexpression, we used lentiviral transduction of LeGO-iG2 vectors (61). As control, hCD34+ cells were transduced with an empty vector construct. Transduced hCD34+ cells were seeded for colony-forming unit assay in MethoCult H4034 Optimum (STEMCELL Technologies) according to the manufacturer’s instructions. Colonies were counted 14 days after seeding. In addition, transduced hCD34+ cells were grown in normal media for 14 days to analyze *in vitro* differentiation by flow cytometry. Antibodies are listed in Table S3.

### Immunofluorescence microscopy

K562 cells were washed in PBS and fixed with 4% paraformaldehyde in PBS for 10 min at 37 °C. Subsequently, the cells were permeabilized with 0.1% Triton X-100 in PBS for 15 min and blocked with 2% bovine serum albumin (BSA) in PBS for 30 min. Samples were incubated overnight at 4 °C with specific primary antibodies diluted in 0.1% BSA in PBS, followed by incubation with AlexaFluor (488, 647)-labeled secondary antibodies, Phalloidin 555 (ThermoScientific) and 4′,6-diamidino-2-phenylindole in in 0.1% BSA in PBS for 45 min at room temperature. Samples were washed in PBS and mounted on coverslip containing Fluoromount-G (SouthernBiotech). The samples were analyzed on an LSM710 confocal laser scanning microscope (Carl Zeiss). Antibodies are listed in Table S4. Negative control is shown in Fig. S1.

### Coimmunoprecipitation

For coimmunoprecipitation, extracts from transiently transfected HEK293T cells were used. HEK293T cells were lysed in lysis buffer (50 mM Tris, pH 7.5, 150 mM NaCl, 10 mM NaF, 20 mM β-glycerophosphate, 0.1% Igepal, 1 mM EDTA, and protease inhibitors). The lysed cells were treated with benzonase (25 U/μl) digest for 2 h rotating at 4 °C. Lysates were centrifuged at 14,000*g* for 10 min, and the supernatants were collected and 800 μg of the protein lysates were incubated in 300 μl lysis buffer containing 50 μl of anti-FLAG magnetic beads (Invitrogen) at 4 °C for 2 h with rotation. The beads were washed two times with lysis buffer, once with ultrapure water. Beads were resuspended in SDS-loading dye. The coimmunoprecipitation samples were analyzed by Western blot. Full blots are given in Fig. S2.

### ChIP assays

ChIP assays were performed according to the X-ChIP protocol (Abcam), with modifications. Sequences of primer pairs used for ChIP–quantitative PCR are given in Table S6. PCR-Primers were located 200 bp upstream of the transcriptional start site; at this region, primer produced comparably specific PCR products. Sonification was performed with a Bioruptor (Diagenode), and average DNA fragments were 400 bp in length in average, allowing coverage of the proximal promoter area. DNA recovery was calculated as percentage of the input. For analysis of the histone modifications, DNA recovery was normalized to a histone H3 ChIP. Antibodies for ChIP are given in Table S9.

### Luciferase reporter assay

For promoter luciferase reporter assays, the 5′-promoter regions of *ID3* were introduced into the pGL4.10 luciferase vector (Promega). Luciferase reporter gene assays were performed as described previously in HEK293T cells (52).

### Gene expression analysis

Total RNA was extracted using the Quick-RNA Mini-Prep-Kit (Zymo-Research) and reverse-transcribed using RevertAid First Strand Cdna Synthesis Kit (ThermoScientific). Quantitative PCR was performed using a SYBR-Green chemistry (PCR-Mastermix, Biozym). PCR values were calculated according to the ΔΔCt quantification method using GAPDH gene as reference for normalization. Sequences of primer pairs are given in Table S5. Genome-wide gene expression analysis was previously performed (32). Hairpin sh-sequences are shown in Table S2. Data were deposited in the Gene Expression Omnibus-Expression database, GSE92251. Further analysis of the candidate genes was performed using the webtool “string” (62) with default settings.

### GST pull-down assay

GST pull-down assays were performed as described previously (52). Proteins were pulled out with glutathione beads (Pierce, Thermo Scientific), detected by SDS-PAGE, and analyzed by autoradiography as described (32). Coomassie stainings as well as full blots are given in Fig. S3.

### Statistics

Experiments were performed at least three times and were statistically analyzed using GraphPad Prism 10 software (GraphPad Software, Inc). Error bars represent the SD from the mean. *p* Values were calculated using the Student’s *t* test. *p* Values < 0.05 were considered statistically significant (∗*p* < 0.05; ∗∗*p* < 0.01; and ∗∗∗*p* < 0.001).

## Data availability

The data underlying this article are within the article, and in the online supporting information, this includes full-length blots. To request data or material from this study, please contact the corresponding author Jörn Lausen (J. L.).

## Supporting information

This article contains supporting information.

## Conflict of interest

The authors declare that they have no conflicts of interest with the contents of this article.
