# Supplementary material for: ID3 promotes erythroid differentiation and is repressed by a TAL1–PRMT6 complex
Source: J Biol Chem. 2024 Dec 22;301(2):108119. doi: 10.1016/j.jbc.2024.108119 (PMC11847539; doi:10.1016/j.jbc.2024.108119)
Supplement: Supporting Information [file mmc1.pdf]

Supporting Information

Heller et al.

Heller et al.

Supplementary Figure S1 related to Figure 1

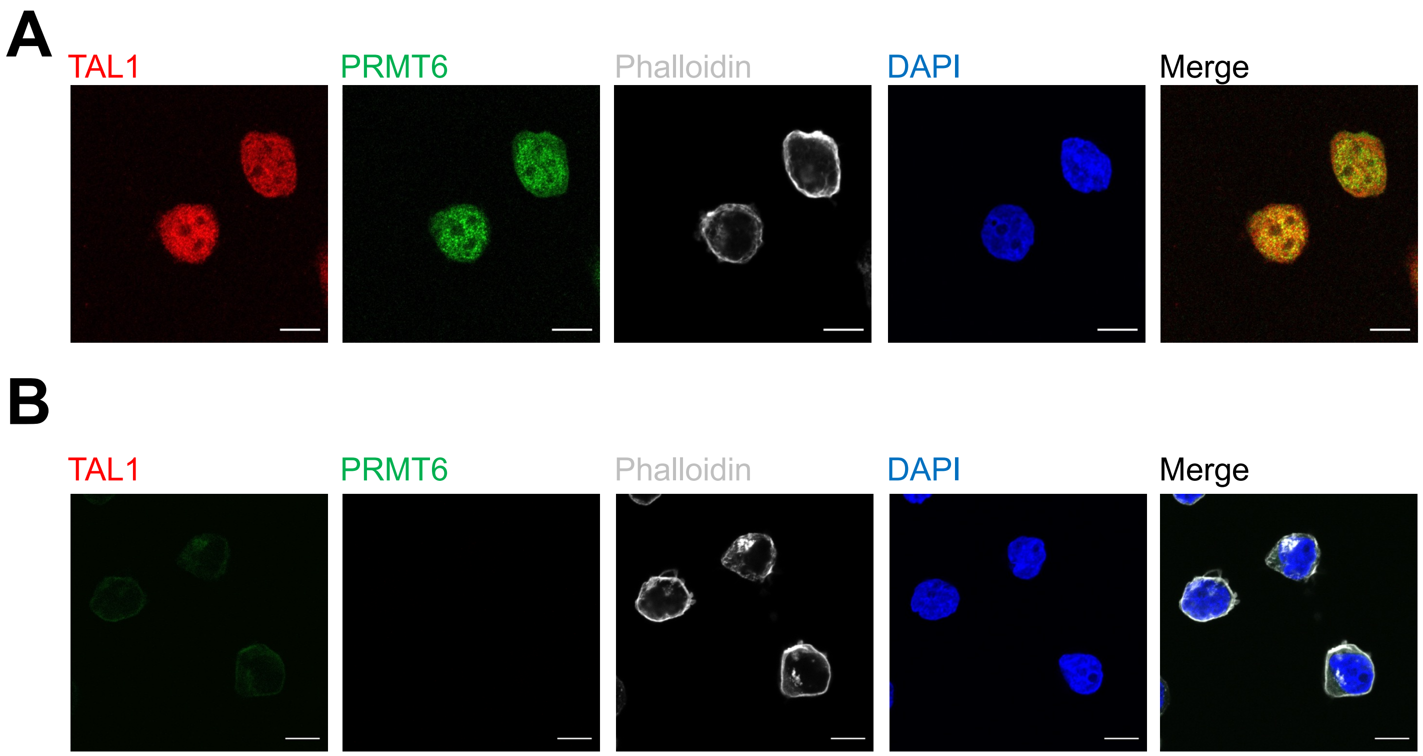

Supplementary Figure S1 related to Figure 1: TAL1 interacts with PRMT6
(A) TAL1 and PRMT6 immunostaining in K562 cells with specific primary antibodies and AlexaFluor (488, 647) labeled secondary antibodies. F-actin-specific immunostainings plus nuclear counterstain (DAPI). **(B)** Negative control for TAL1 and PRMT6 immunostaining in K562 cells. Only primary antibodies were used and no AlexaFluor (488, 647) labeled secondary antibodies. F-actin-specific immunostainings plus nuclear counterstain (DAPI). The samples were analyzed on a LSM710 confocal laser scanning microscope (Carl Zeiss, Oberkochen, Germany) equipped with a Plan-Apochromat 63x/1.40 DIC (Carl Zeiss) oil immersion objective.
Images are maximum intensity projections of several confocal sections (ZEN software, Zeiss). Scale bars: 10 µm.

Heller et al.

Supplementary Figure S2 related to Figure 1

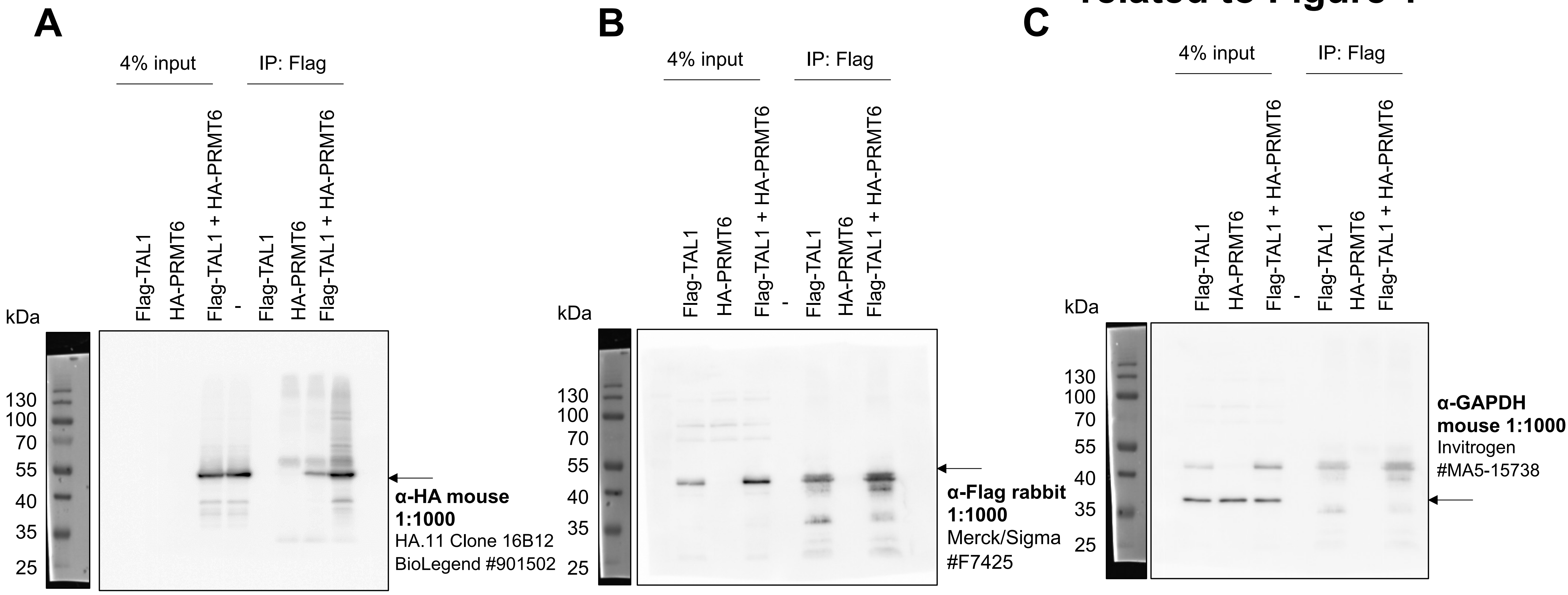

Supplementary Figure S2 related to Figure 1: TAL1 interacts with PRMT6

(A-C) Co-immunoprecipitation of overexpressed HA-tagged PRMT6 and FLAG-tagged TAL1 in HEK293T cells. Lysates were incubated with Anti-FLAG magnetic beads and analyzed via SDS-PAGE and Western Blotting by using HA-or FLAG-specific primary antibodies. Secondary antibody anti-rabbit or anti-mouse IgG H&L (HRP) (Abcam, ab97080) was used with a dilution of 1:10.000. Antibodies were diluted in 4% milk/TBS-T. GAPDH was used as loading control. Raw data are depicted in this figure. Marker: PageRuler Prestained #26617 (Invitrogen).

Heller et al.

Supplementary Figure S3 related to Figure 1

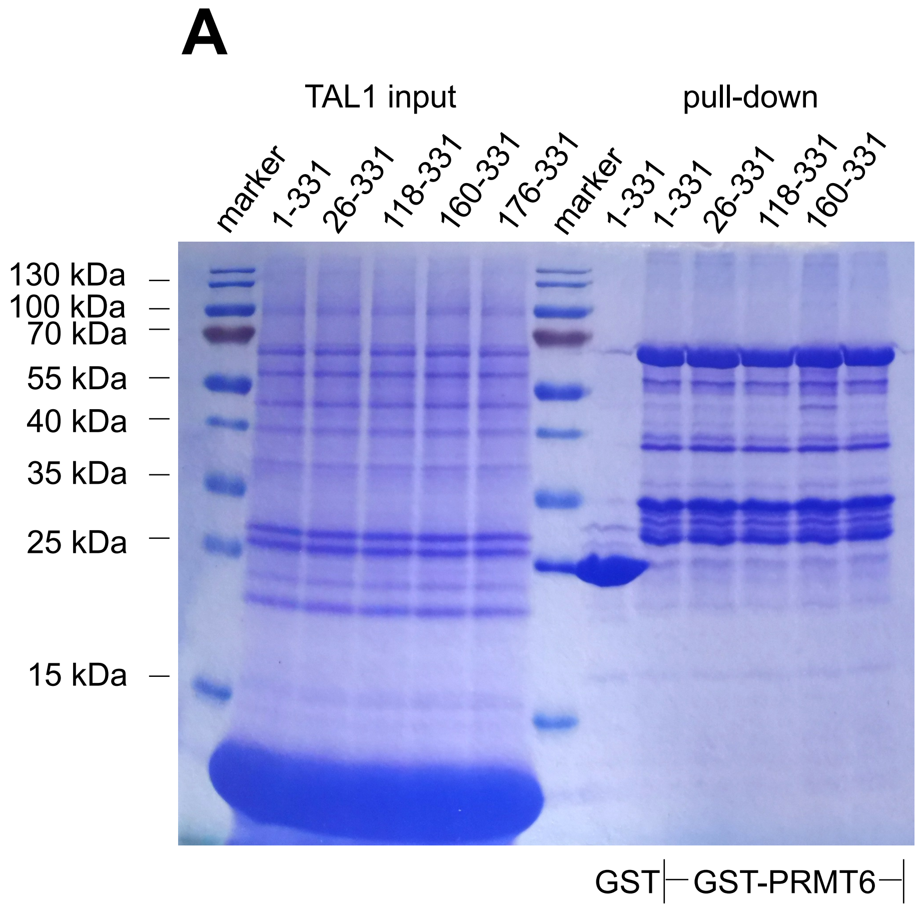

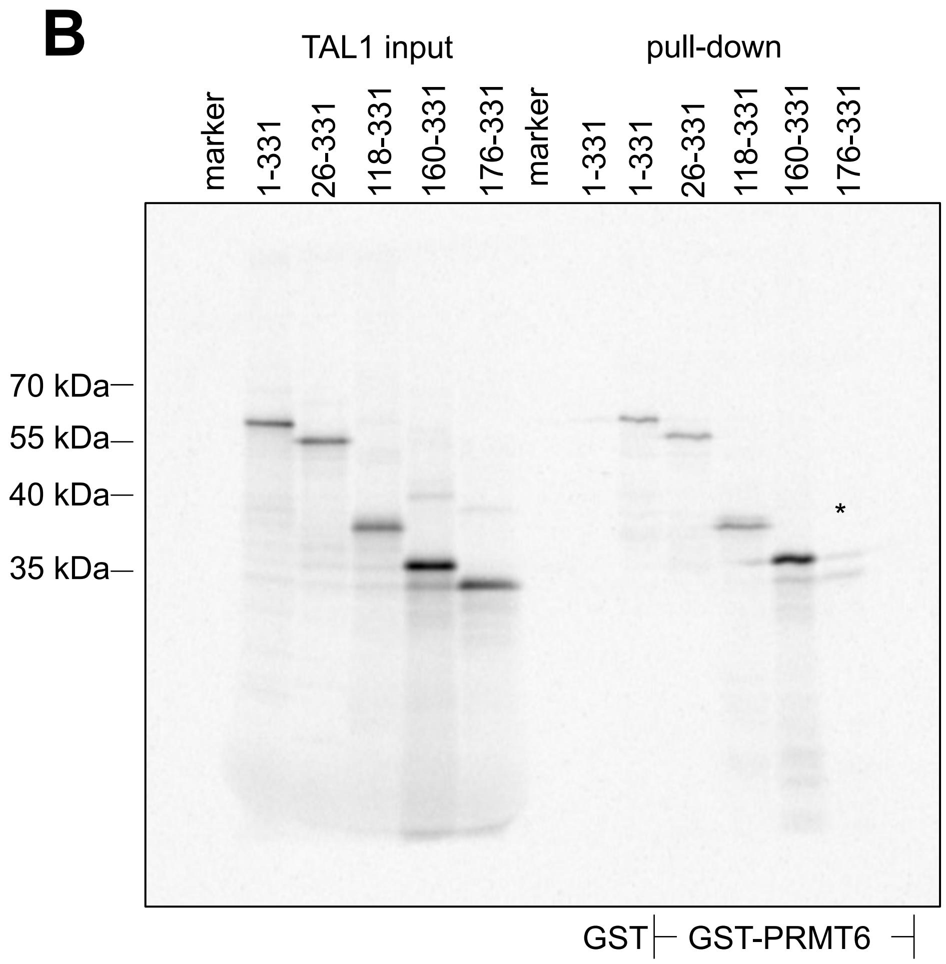

Supplementary Figure S3 related to Figure 1

C

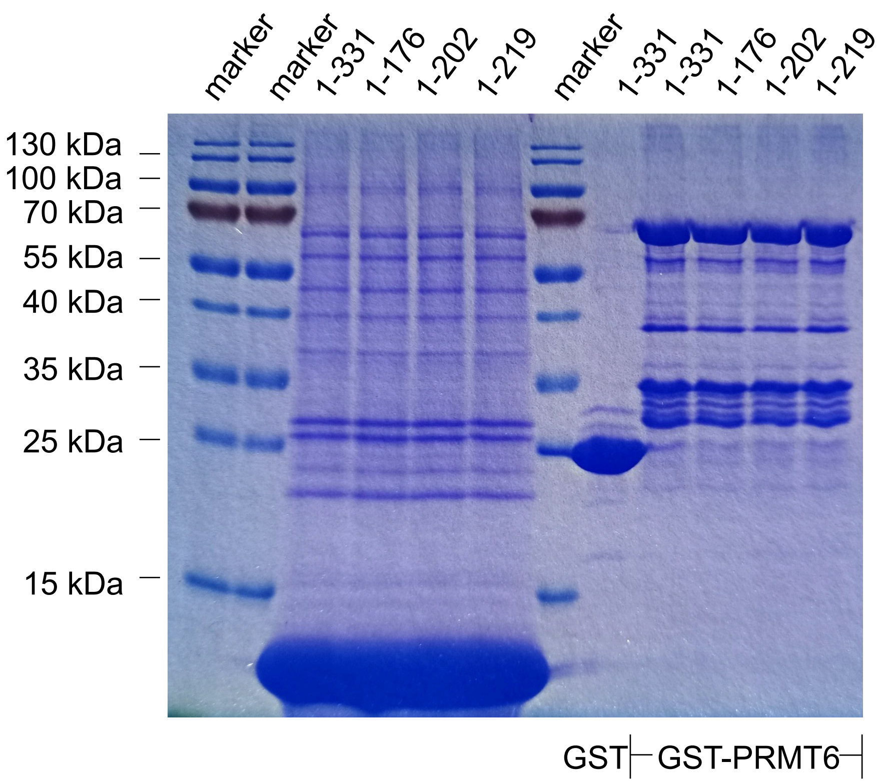

D

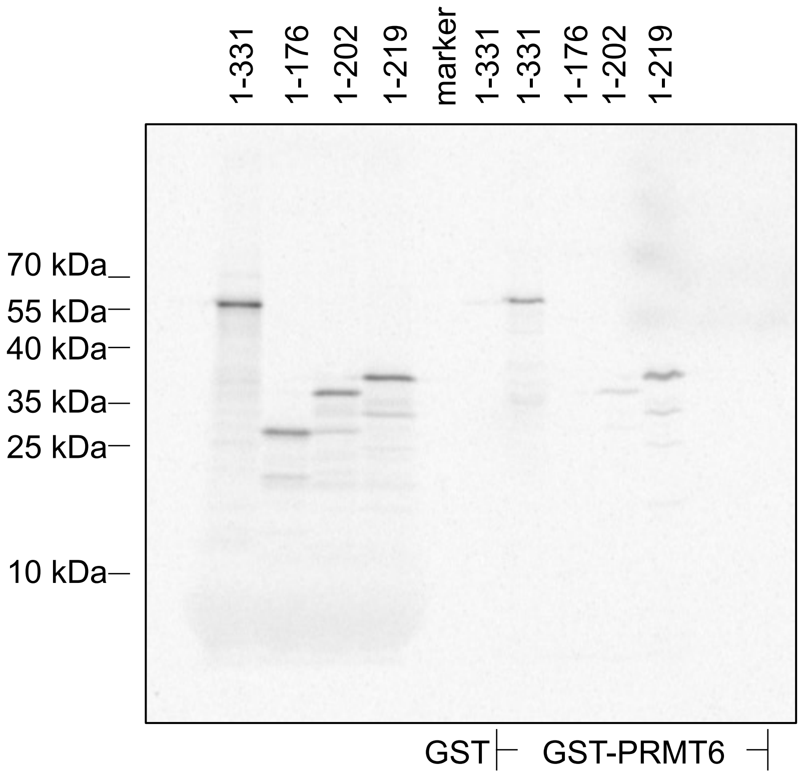

Heller et al.

Supplementary Figure S3 related to Figure 1

E

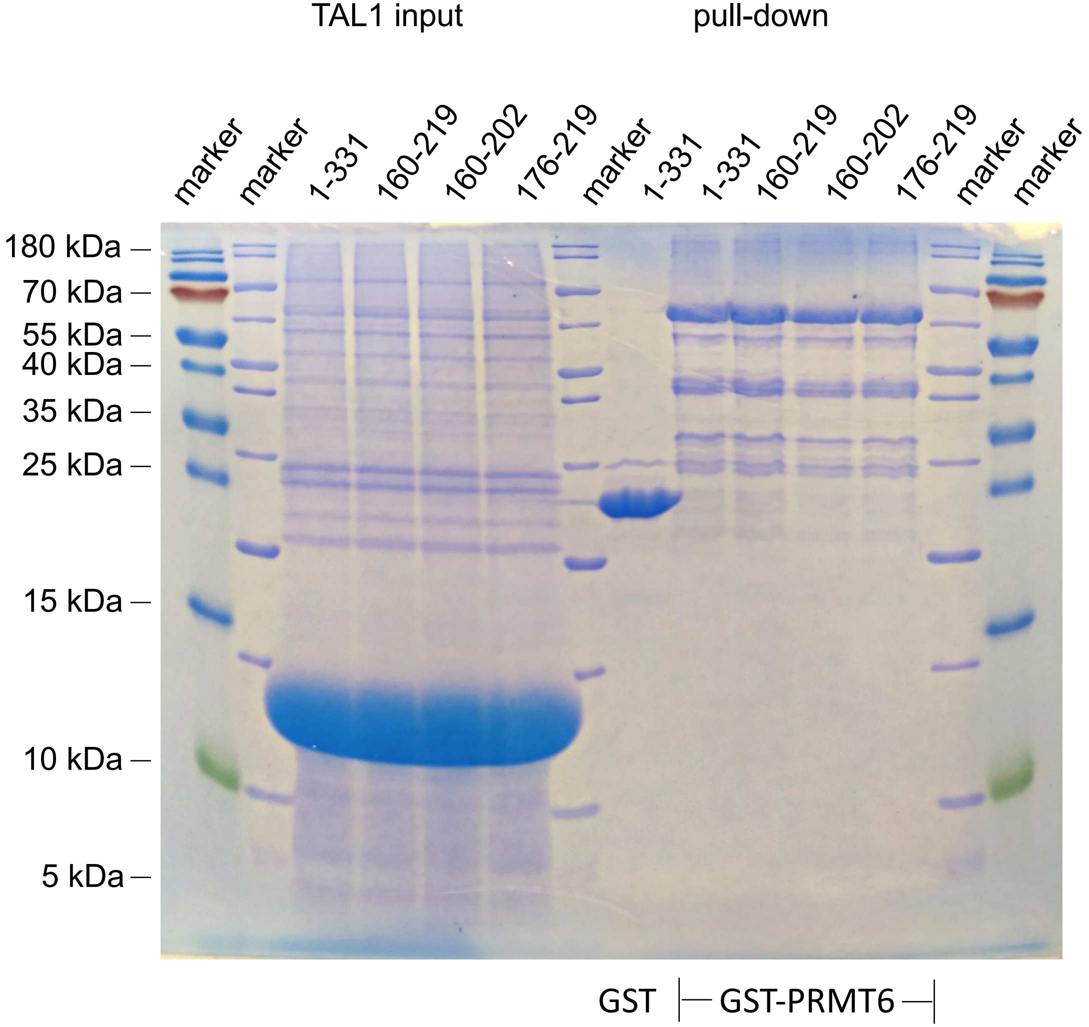

F

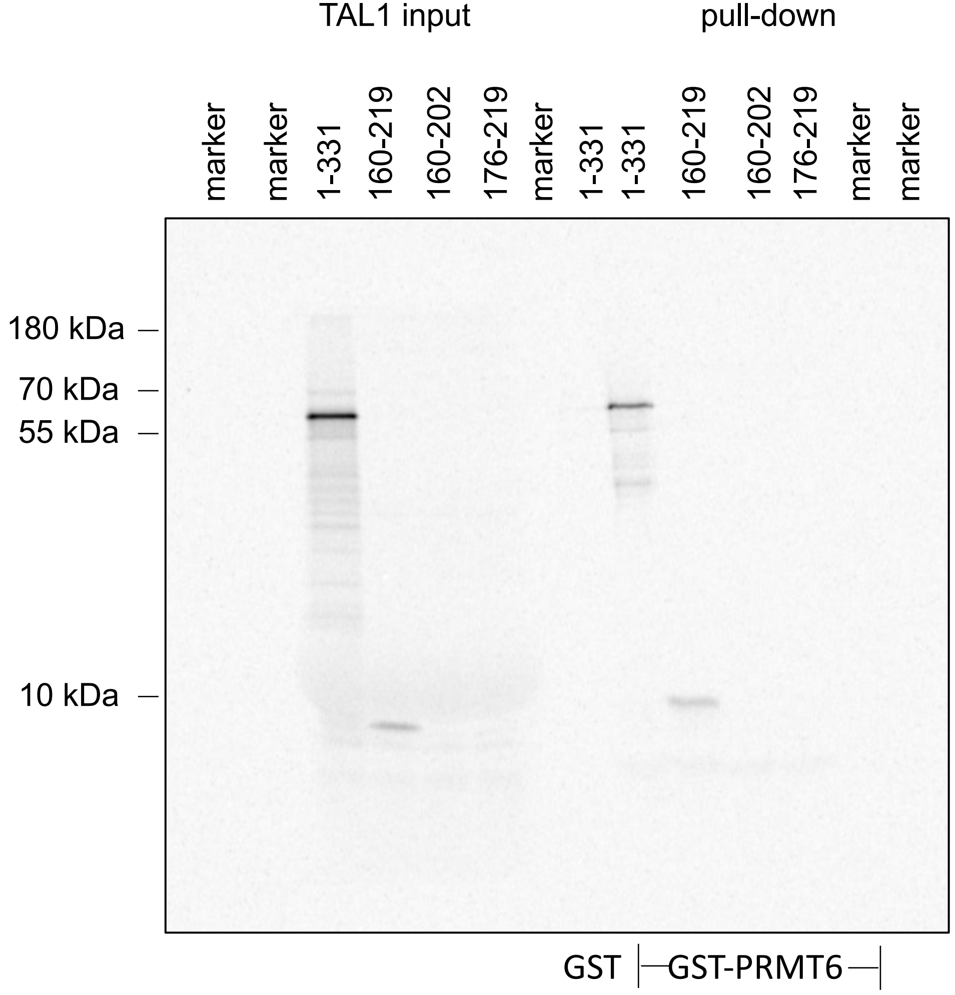

Supplementary Figure S3: TAL1 interacts with PRMT6
(A,C,E) Coomassie staining of the gels for mapping the interaction domain of TAL1 with PRMT6 using a GST pulldown: GST-PRMT6 protein was expressed in E. coli, purified and incubated with different in vitro transcribed/translated 35S-methionine marked TAL1 constructs (numbers refer to aminoacid position). In (E) only TAL1 aa160-219 was used because in vitro transcription/translation did not work for other constructs. **(B,D,F)** Uncropped radioautographied GST pulldowns. Marker: PageRuler Prestained #26617 (Invitrogen).

Heller et al.

Supplementary Figure S4 related to Figure 2

Gene name	shTAL1	shPRMT6
A2M	-2,53	1,01
ABCC3	1,17	1,60
ABCC6	1,42	-1,54
ACVRL1	2,32	1,85
AHSP	-1,89	1,56
AIM1	2,12	-1,06
AIM2	2,51	-1,36
AMIGO2	1,32	-1,07
ANKRD35	1,34	1,19
ANKRD55	1,95	-1,46
ANUBL1	-1,96	-1,55
ARL4C	-1,47	-1,15
ASS1	-1,44	-2,10
ATG16L2	1,09	1,26
ATP8A2	2,01	1,70
BCL6	1,56	1,13
BDNF-AS1	1,53	1,82
BTNL3	1,04	1,31
CA1	-3,53	1,95
CAB39L	1,08	-1,30
CALB1	3,04	-1,75
CALCOCO1	1,35	1,53
CAMKV	-1,04	-2,56
CCDC149	-1,51	-1,34
CD24	1,59	-2,29
CD84	2,37	1,01
CDH23	-3,37	-2,88
CFB	1,57	1,03
CHST3	-1,90	-1,80
CLC	-1,16	-1,28
CLIC3	2,34	1,40
CLU	2,71	1,25
CPZ	1,29	-2,06
CRTC1	-1,11	1,37
CXCL1	1,46	-2,55
CXCL3	1,03	-1,21
CXCR4	-1,63	-1,12
CYP27A1	-1,39	-1,19
CYP3A7	1,04	1,14
CYTH4	3,26	1,35
DBNL	1,32	1,46
DCBLD2	1,34	1,46
ECM1	1,93	1,51
EFHB	1,06	1,26
ENTPD1	1,22	1,16
ENTPD8	1,13	1,01
EPB42	-2,64	1,40
ESR2	-1,28	-2,05
FAM122C	-1,07	1,27
FAM23A	-1,24	1,19
FAM47E	1,50	-2,30
FCRLA	3,02	1,37
FJX1	-1,27	-3,20
FLJ31104	1,25	1,51
FLJ46120	1,98	1,12
FMO4	1,42	-1,42
FRY	1,66	1,45
GBP2	1,77	1,21
GCH1	-1,14	-1,07
GDF1	-2,50	-1,38
GFPT2	-1,08	1,84
GIMAP2	1,61	-1,26
GPR183	2,39	-1,29
GSN	1,53	1,27
HGD	1,41	1,01
HPGD	1,49	-2,23
HRK	-1,83	-1,03
HSD11B1	4,01	1,94
HSD11B2	-1,12	-1,25
HTR4	1,65	1,04
ID2	1,16	1,11
ID3	2,32	1,35
IGFL2	1,97	-2,18
IKZF2	1,48	-1,32
IL10RA	1,32	-1,66
IL2RB	1,25	-1,15
IRF6	2,55	-1,10
ITGA4	-1,34	-1,10
KALRN	-1,52	-2,34
KIAA1683	1,23	1,56
KIF7	-1,57	-1,47
KIT	1,37	-2,76
KLHDC8A	-1,60	1,04
KRT79	1,41	1,09
LAMB3	2,28	1,99
LASS1	-2,43	-1,71
LGALS3	1,61	2,04
LYVE1	1,25	-1,80
LYZL4	2,74	1,17
MARCKS	1,10	-2,66
MGC12916	1,53	-1,73
MGP	1,11	1,36
MPP7	-1,22	-1,02
MSMB	1,14	-1,01
MTSS1	1,39	1,03
MYO1F	1,55	1,16
MYO5A	1,39	-1,68
NAAA	1,05	-1,43
NAT8L	-1,57	-2,51
NBL1	-1,30	-1,48
NCRNA00261	2,54	-3,00
NOX5	2,56	1,57
NQO1	1,04	-1,09
NRG1	-1,93	1,28
NRP1	1,51	-1,37
OR6C76	1,07	1,88
P2RX1	2,20	1,08
PALLD	-1,23	-2,61
PDE4DIP	1,02	-1,79
PLA2G3	1,07	-1,39
PNRC1	1,28	1,36
POLD4	1,23	1,06
PPFIBP1	1,35	-1,13
PPOX	-1,07	1,05
PRDM1	1,50	1,08
PTPN22	1,04	-1,30
RAB27B	1,11	-1,35
RAB7B	1,60	1,26
RASGEF1A	-1,02	-1,21
RASSF4	2,43	1,06
RDH10	1,06	-1,52
RGS16	1,31	-1,46
RHBDF2	1,00	-1,08
RNASET2	1,34	-1,12
RPS6KA2	1,24	-1,88
RSAD2	1,58	1,73
RUNX2	1,81	-1,13
S100P	-1,11	-1,31
S1PR1	4,34	-1,61
SCARF2	-1,13	1,20
SCN9A	1,67	-2,64
SDPR	1,33	-1,20
SGK223	1,55	1,01
SH3BP2	1,94	-1,37
SLC22A11	1,58	-2,52
SLC24A2	2,08	-1,31
SLC25A37	-1,20	1,21
SLC2A3	1,79	-1,44
SLC45A3	1,05	1,23
SLFN11	-1,41	-1,17
SLITRK6	1,92	-1,34
SOSTDC1	2,01	-2,19
SPARC	1,16	1,48
SSC5D	1,23	1,26
SULT1C2	-1,61	-1,56
TCEAL6	-2,02	1,98
TGFBI	1,48	1,01
TMEM158	1,35	-1,08
TMPRSS11F	1,56	1,37
TNFAIP8L2	1,08	-1,02
TNRC18	1,33	1,27
TNS1	-2,36	1,29
TP53INP1	2,10	1,45
TPP1	1,03	1,03
TRPV1	-1,24	1,19
USP18	-1,03	-1,21
WBSCR27	1,26	1,26
YPEL3	1,25	1,75
ZC3H6	1,08	1,63
ZFP42	-1,24	-1,42

Supplementary Figure S4: TAL1 and PRMT6 have 160 common target genes.
(A) Knockdown of TAL1 or PRMT6 in K562 cells. Gene expression changes upon knockdown compared to the control are given in logFC. In about 65% of all upregulated genes TAL1 binding in the blood system could be found in proximity of the gene. This indicates that potentially a substantial number of genes are repressed by TAL1.

Heller et al.

Supplementary Figure S5 related to Figure 2

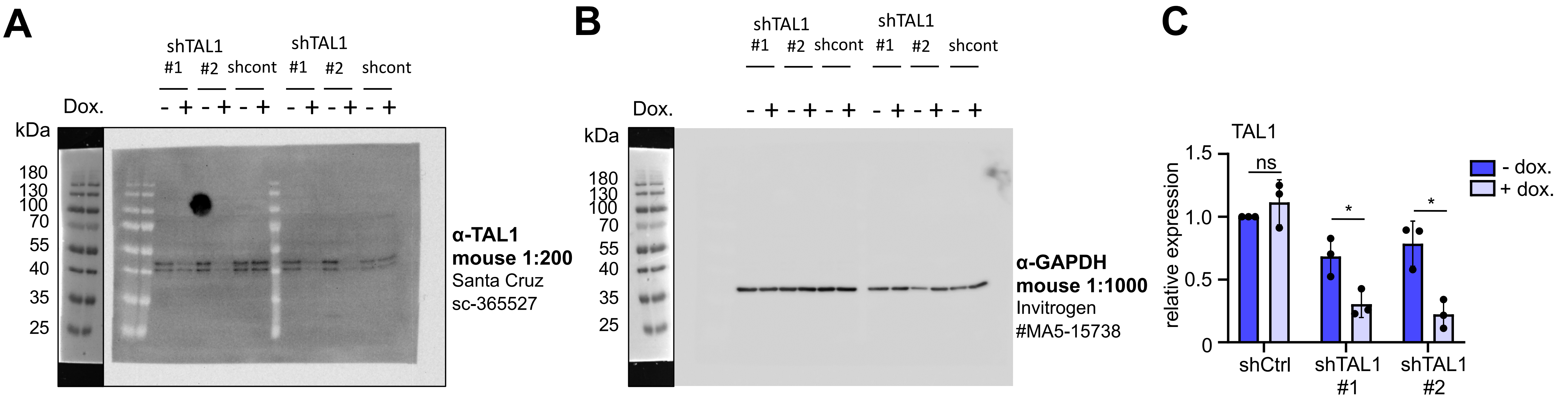

Supplementary Figure S5: TAL1 and PRMT6 have common target genes.
(A, B) Stable inducible TAL1 knockdown was established. Western blot shows knockdown of TAL1 in K562 upon induction with 1 µM doxycycline. GAPDH serves as loading control. Secondary antibody anti-rabbit or anti-mouse IgG H&L (HRP) (Abcam, ab97080) was used with a dilution of 1:10.000. Antibodies were diluted in 4% milk/TBS-T. Detection with ECL. Raw data are depicted in this figure. Marker: PageRuler Prestained #26617 (Invitrogen). Western Blots show two biological replicates.**(C)** TAL1 expression was decreased upon TAL1 knockdown after 7 days. TAL1 expression was determined by RT-qPCR and normalized to GAPDH expression. Graphs show the means ± standard deviation (SD) of three independent experiments (n=3). P-values were calculated using Student t-test (*P < 0.05; **P < 0.01; ***P < 0.001).

Supplementary Figure S6 related to Figure 2

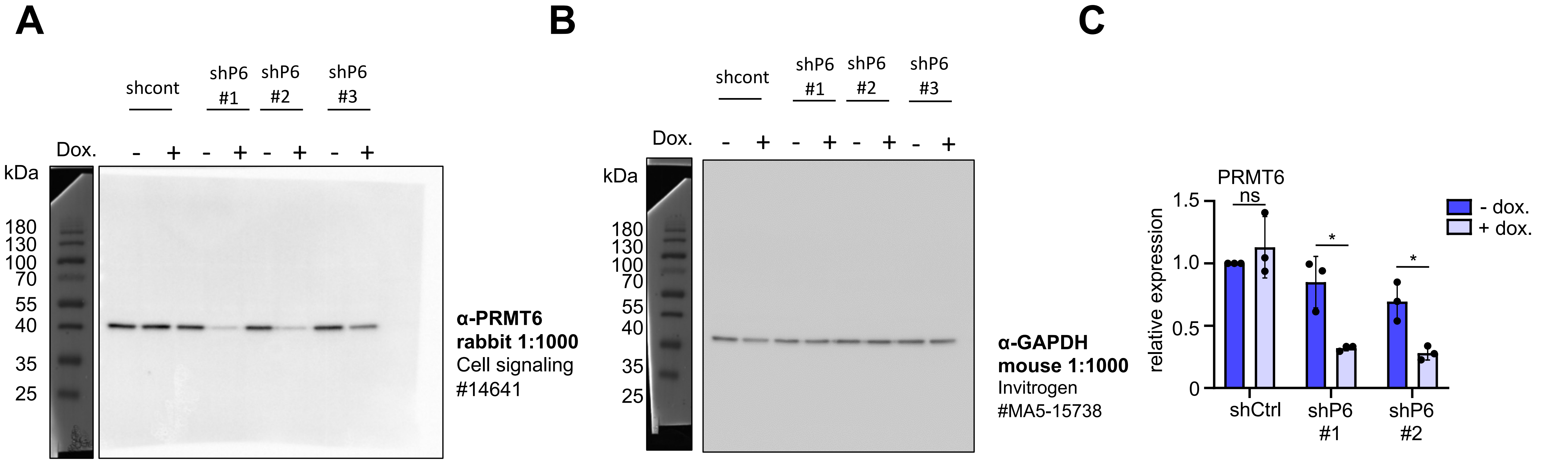

Supplementary Figure S6: TAL1 and PRMT6 have common target genes.

(A, B) Stable doxycycline-inducible PRMT6 knockdown was established. Western blot shows knockdown of PRMT6 in K562 upon induction with 1 µM doxycycline. GAPDH serves as loading control. shP6=shPRMT6. Secondary antibody anti-rabbit or anti-mouse IgG H&L (HRP) (Abcam, ab97080) was used with a dilution of 1:10.000. Antibodies were diluted in 4% milk/TBS-T. Detection with ECL. Raw data are depicted in this figure. Marker: PageRuler Prestained #26617 (Invitrogen). **(C)** PRMT6 expression was decreased upon PRMT6 knockdown after 7 days. PRMT6 expression was determined by RT-qPCR and normalized to GAPDH expression. Graphs show the means ± standard deviation (SD) of three independent experiments (n=3). P-values were calculated using Student t-test (*P < 0.05; **P < 0.01; ***P < 0.001).

Supplementary Figure S7 related to Figure 2

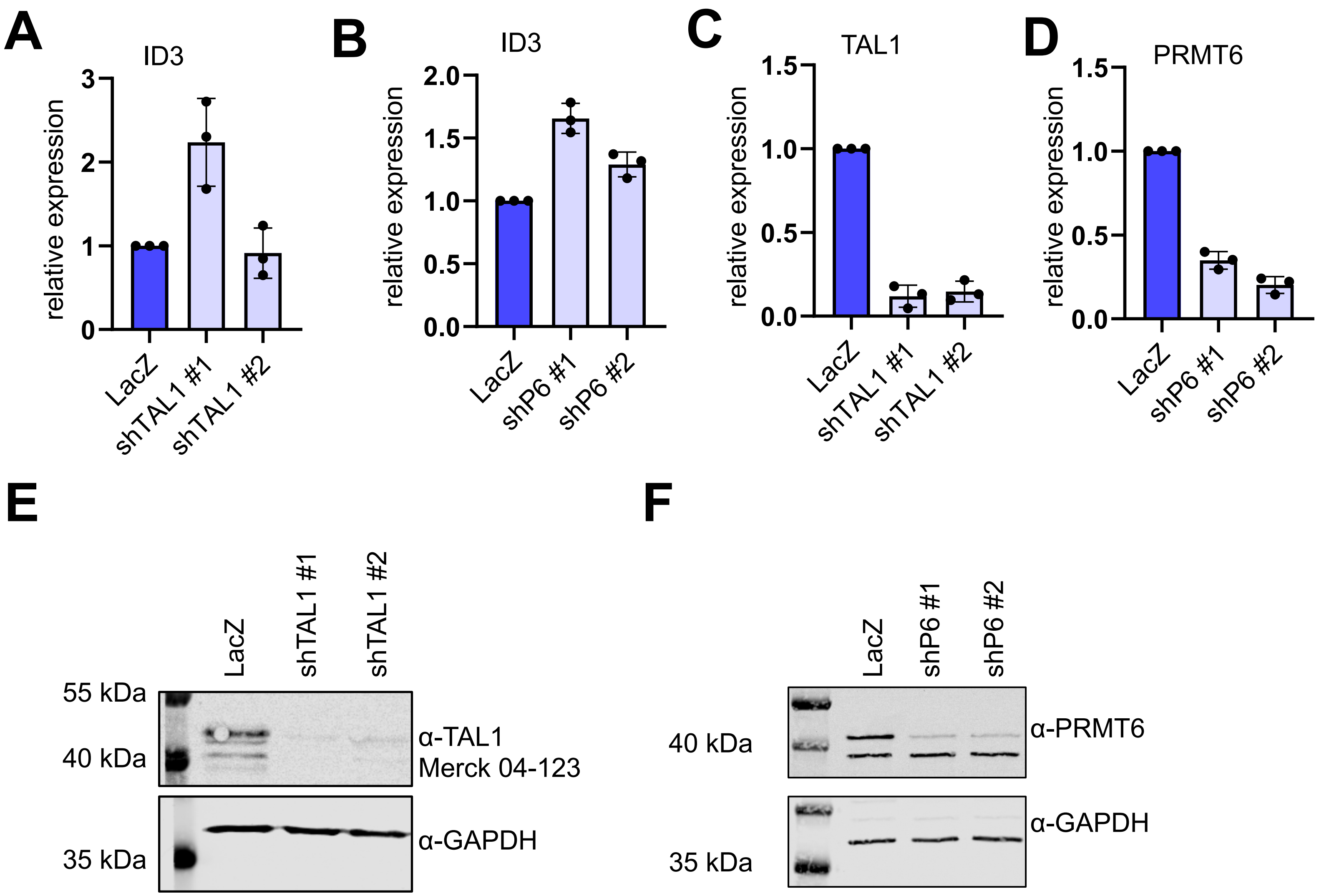

Supplementary Figure S7: TAL1 and PRMT6 have common target genes.
(A) ID3 expression was increased after TAL1 knockdown for one shRNA. **(B)** ID3 expression was increased after knockdown of PRMT6 (shP6). **(C)** Knockdown of TAL1 with SEW vector in K562 cells for 6 days. **(D)** Knockdown of PRMT6 (P6) with SEW vector in K562 cells for 6 days. Expression levels were determined by RT-qPCR and normalized to GAPDH expression. Graphs show the means ± standard deviation (SD) of three independent experiments (n=3). **(E)** Western blot shows TAL1 knockdown in K562 cells after 6 days. **(F)** PRMT6 knockdown in K562 cells after 6 days. GAPDH serves as loading control. shP6=shPRMT6.

Supplementary Figure S8 related to Figure 3

A

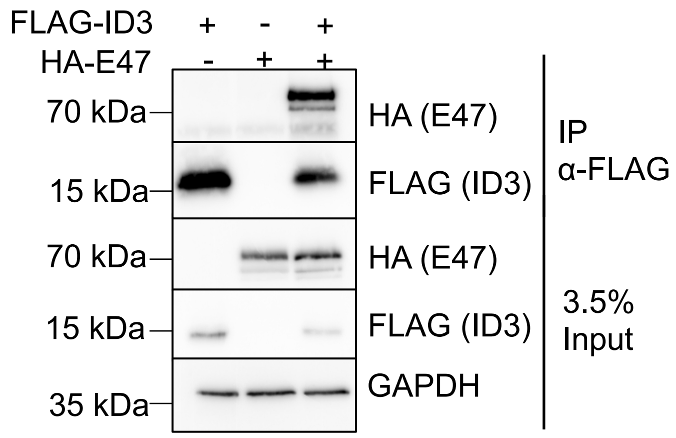

B

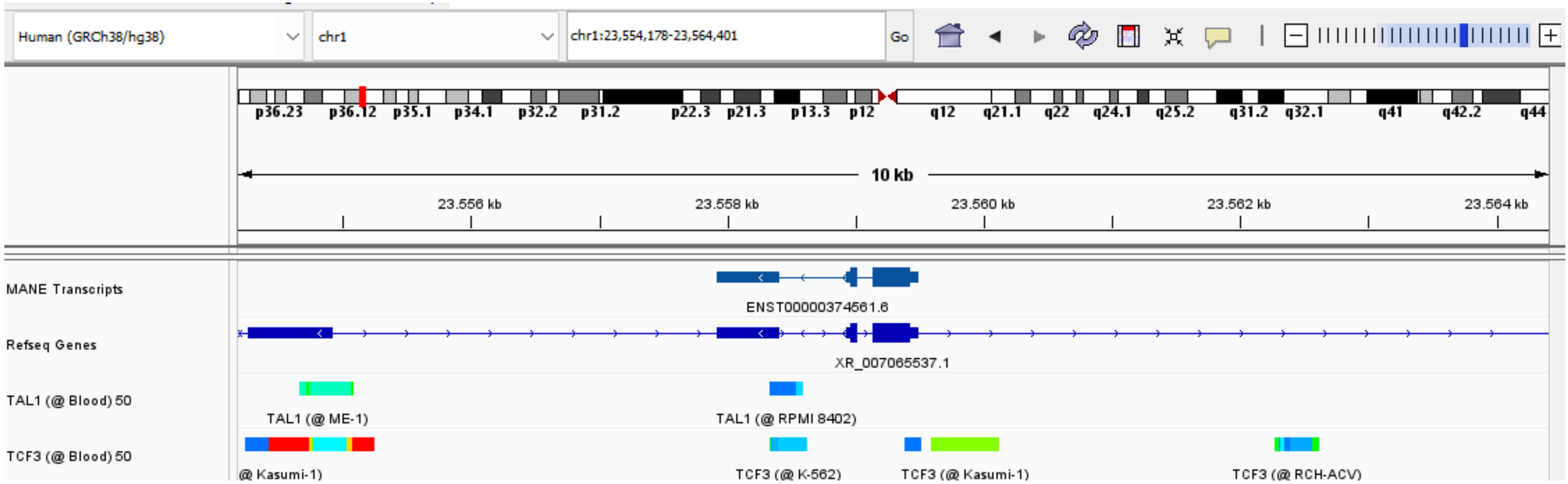

20.02.24

https://chip-atlas.org/peak_browser

Supplementary Figure S8: TAL1, E47 and PRMT6 bind to the ID3 promoter. (A) Co-immunoprecipitation with HA-labeled E47 and FLAG-labeled ID3. Lysates were incubated with anti-FLAG magnetic beads and analyzed by SDS-PAGE and Western blot analysis. **(B)** Screenshot from ChIP atlas (https://chip-atlas.org). TAL1 and E47 (TCF3) were enriched at the ID3 locus in different ChIP sequencing data sets of different cell types.

Heller et al.

Supplementary Figure S9 related to Figure 3

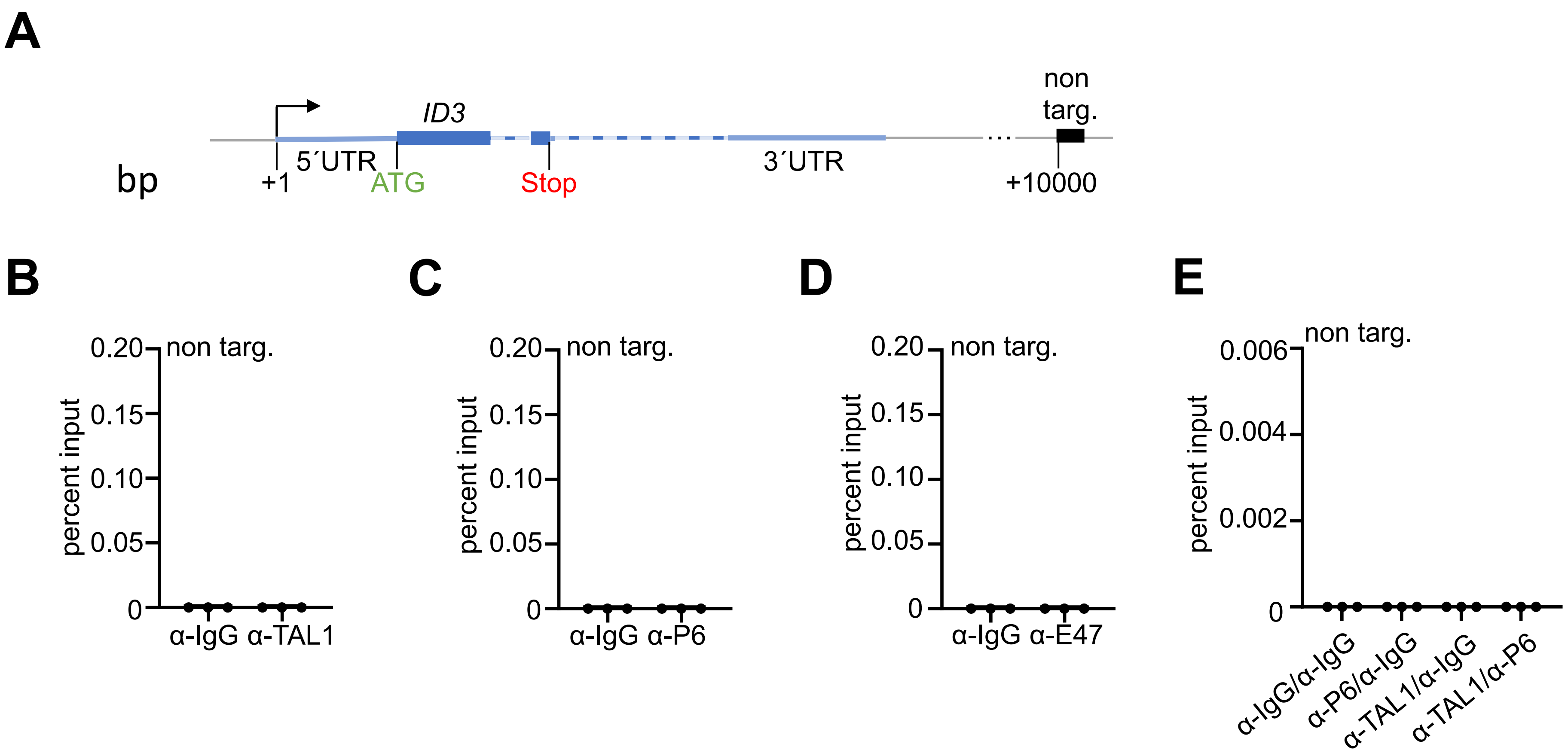

Supplementary Figure S9: TAL1, E47 and PRMT6 bind to the ID3 promoter. (A) Schematic representation of the ID3 gene and its promoter region. The position of the ChIP primers for non targeting (non targ.) control is given (black box). ATG, start codon; bp, base pairs; +1 transcription start point; Stop, stop codon; UTR, untranslated region. **(B-D)** TAL1, E47, and PRMT6 do not bind to the negative control region in K562 cells. ChIP assay quantified via qPCR with specific oligos for the non targeting control region (non targ.). Graphs show the means ± standard deviation (SD) of three independent experiments (n=3). P-values were calculated using Student t-test (*P < 0.05; **P < 0.01; ***P < 0.001). **(E)** ChIP-reChIP analysis of TAL1 and PRMT6 in K562 cells for negative control region. ChIP assay with PRMT6-specific antibodies was performed after TAL1 ChIP. Data are given as percent input. Graphs show the means ± standard deviation (SD) of three independent experiments (n=3).

Heller et al.

Supplementary Figure S10 related to Figure 4

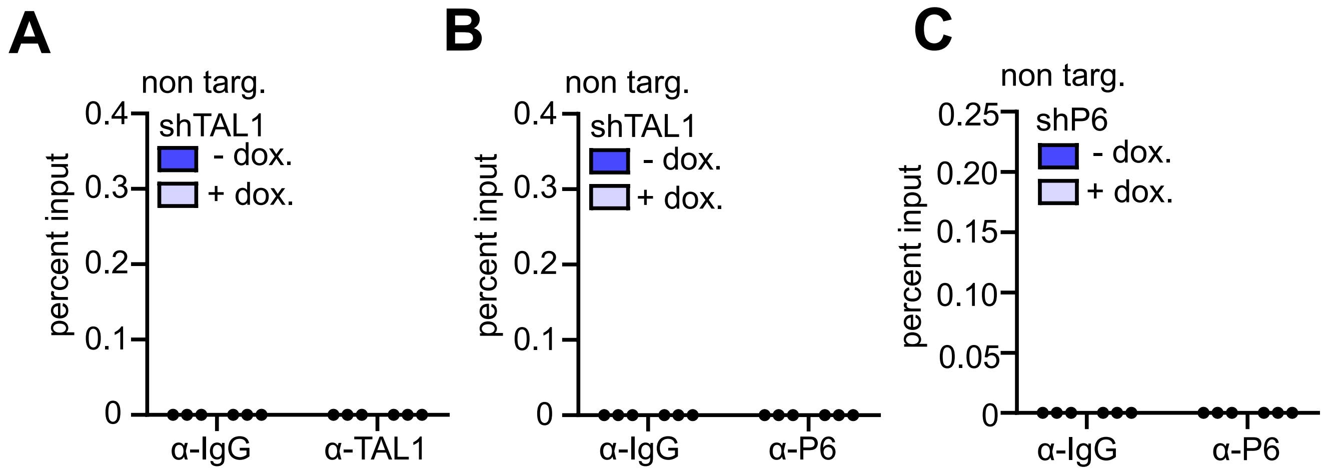

Supplementary Figure S10:TAL1 recruits PRMT6 to the ID3 promoter to set repressing histone modifications. (A) TAL1 does not bind to the negative control region in K562 cells after knockdown of TAL1. **(B, C)** PRMT6 does not bind to the negative control region in K562 cells after knockdown of TAL1 or PRMT6. P6, PRMT6; dox; doxycycline. ChIP assay quantified via qPCR with specific oligos for the non targeting control region (non targ.). Graphs show the means ± standard deviation (SD) of three independent experiments (n=3). P-values were calculated using Student t-test (*P < 0.05; **P < 0.01; ***P < 0.001). Data are given as percent input. Graphs show the means ± standard deviation (SD) of three independent experiments (n=3).

Supplementary Figure S11 related to Figure 5

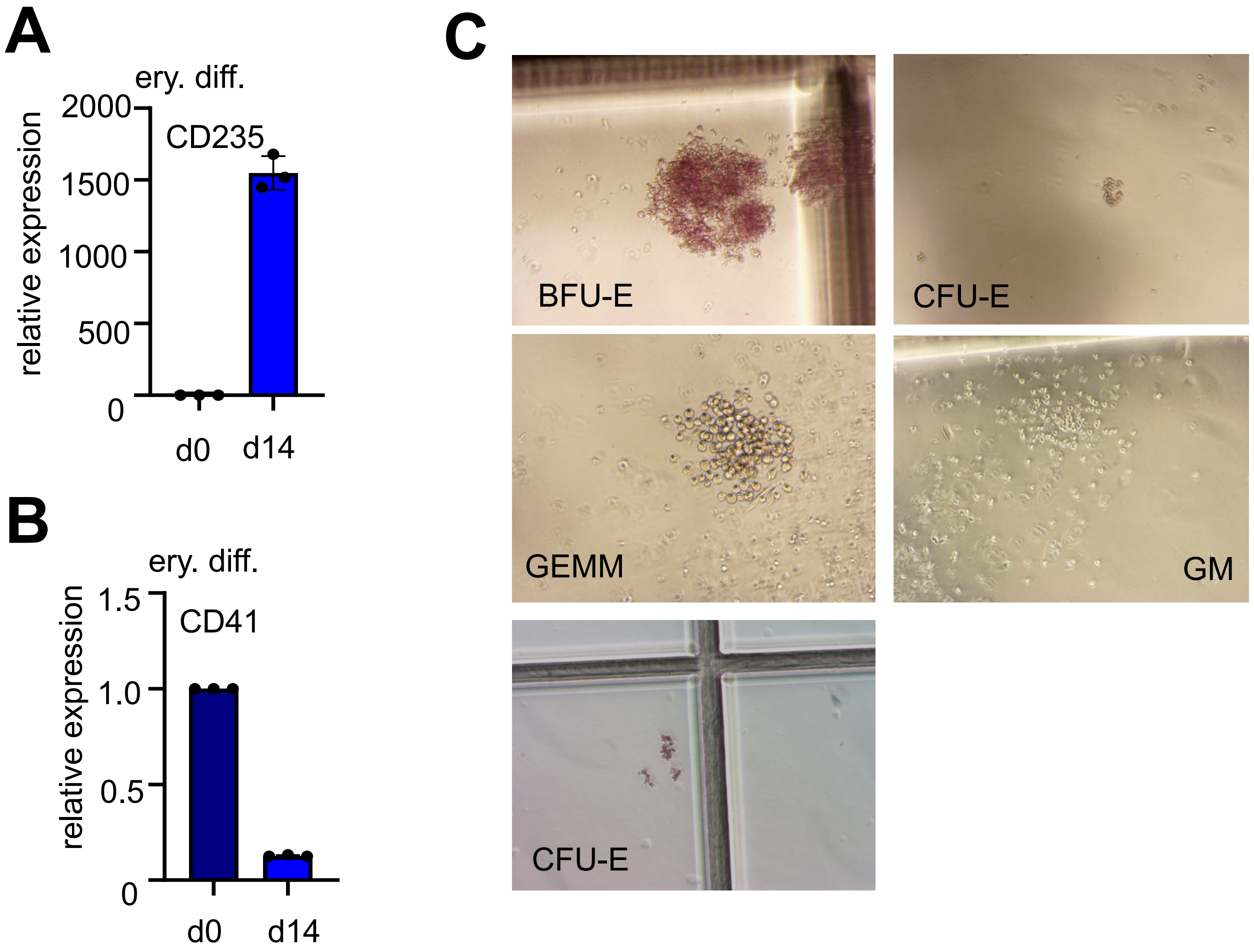

Supplementary Figure S11: ID3 overexpression leads to erythroid differentiation. (A) Expression of CD235 was increased after 14 days of induction with erythroid supplements (StemCell Technologies) in CD34+ cells. **(B)** Expression of CD41 was decreased. Expression levels were measured on the mRNA level by RT-qPCR and normalized to GAPDH expression. The relative expression of undifferentiated CD34+ cells are set as one. Graphs show the means ± standard deviation (SD) of three independent experiments (n=3). **(C)** Pictures taken with microscope during counting of CFU. CFU-E, colony-forming unit-erythroid; BFU-E, burst-forming unit-erythroid; CFU-GM, granulocyte–macrophage progenitor; CFU-GEMM, common myeloid progenitor. Microscope objectives: 10x, 4x (only for picture on the bottom left).

Heller et al.

Supplementary Figure S12 related to Figure 5

A

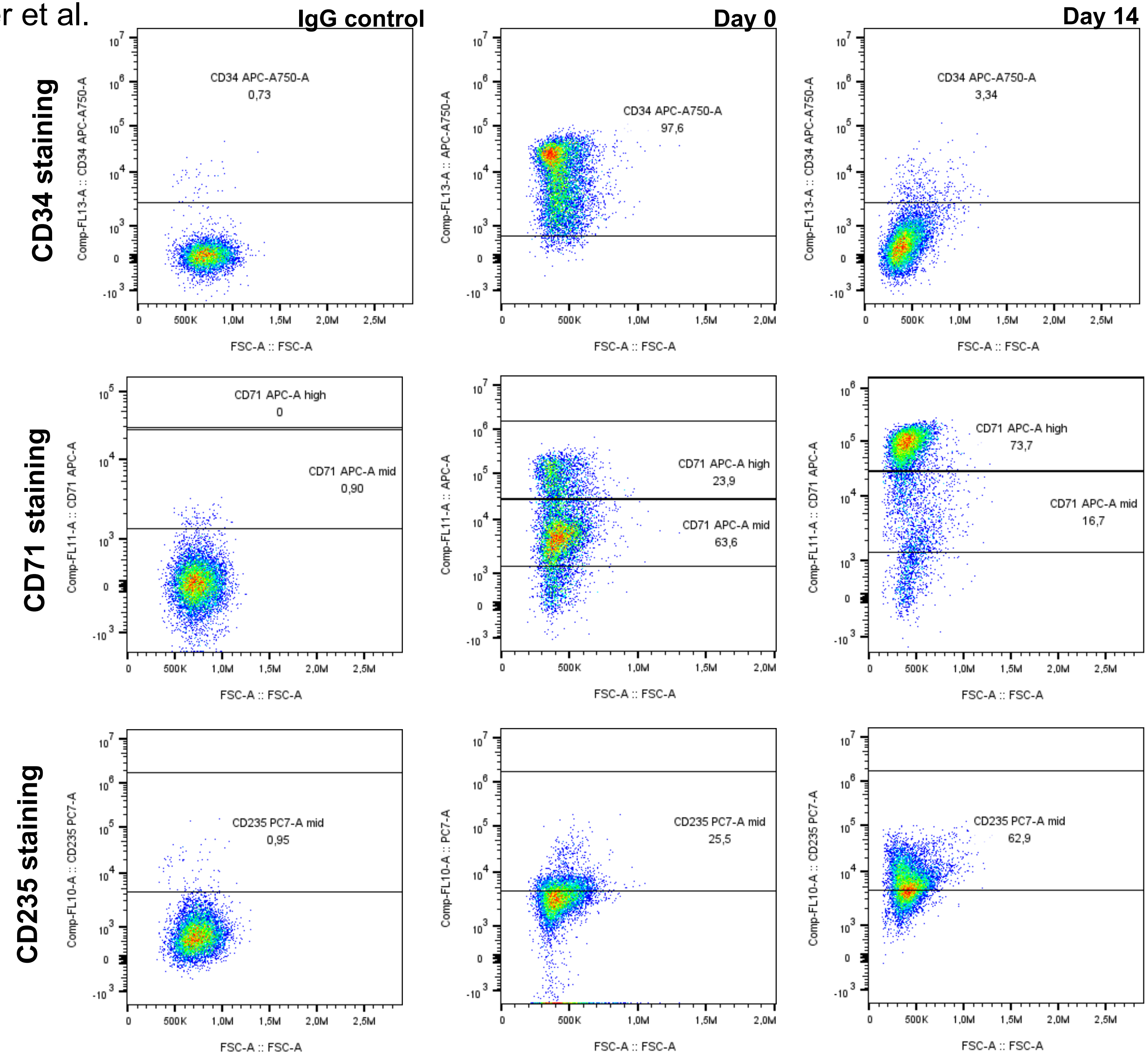

Supplementary Figure S12: ID3 overexpression leads to erythroid differentiation. (A) Differentiation of hCD34+ cells was monitored by flow cytometry. For CD34 staining IgG control, day 0 and day 14 is shown. CD34 was decreased upon differentiation to erythrocytes. CD71 was increased after 14 days of differentiation, as well as CD235. **(B)** Flow data shows GFP signal on day 0 and day 14 for control and ID3 overexpressing cells. Vector used in this experiment is LeGO IG2, which contains GFP cassette. **(C)** Flow data corresponding to bar chart. CD235 staining and CD71 staining is shown. Day 0 compared to day 14 for hCD34+ cells control and ID3 overexpressing ones.

Supplementary Figure S12 related to Figure 5

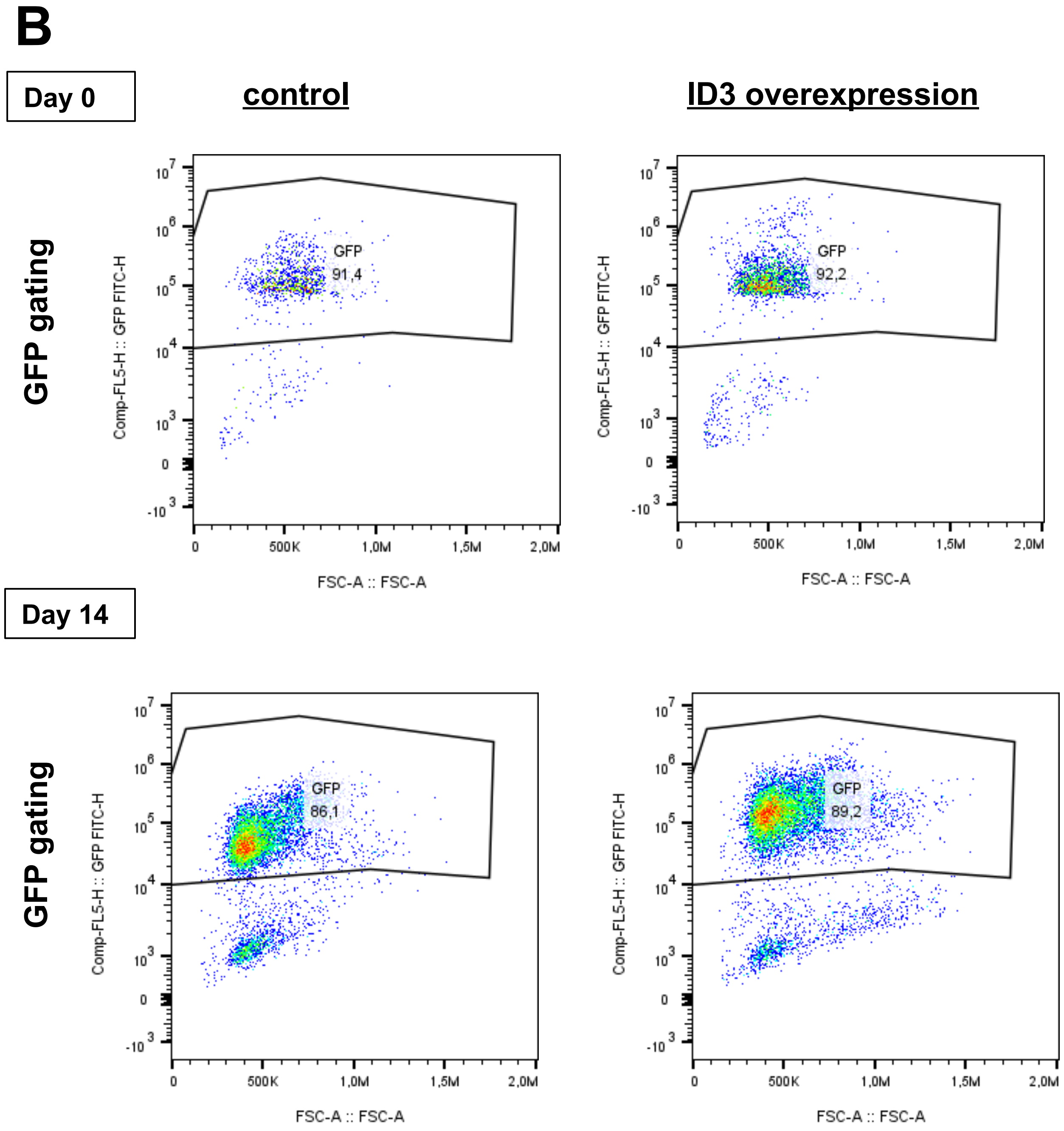

Supplementary Figure S12: ID3 overexpression leads to erythroid differentiation. (A) Differentiation of hCD34+ cells was monitored by flow cytometry. For CD34 staining IgG control, day 0 and day 14 is shown. CD34 was decreased upon differentiation to erythrocytes. CD71 was increased after 14 days of differentiation, as well as CD235. **(B)** Flow data shows GFP signal on day 0 and day 14 for control and ID3 overexpressing cells. Vector used in this experiment is LeGO IG2, which contains GFP cassette. **(C)** Flow data corresponding to bar chart. CD235 staining and CD71 staining is shown. Day 0 compared to day 14 for hCD34+ cells control and ID3 overexpressing ones.

Supplementary Figure S12 related to Figure 5

C

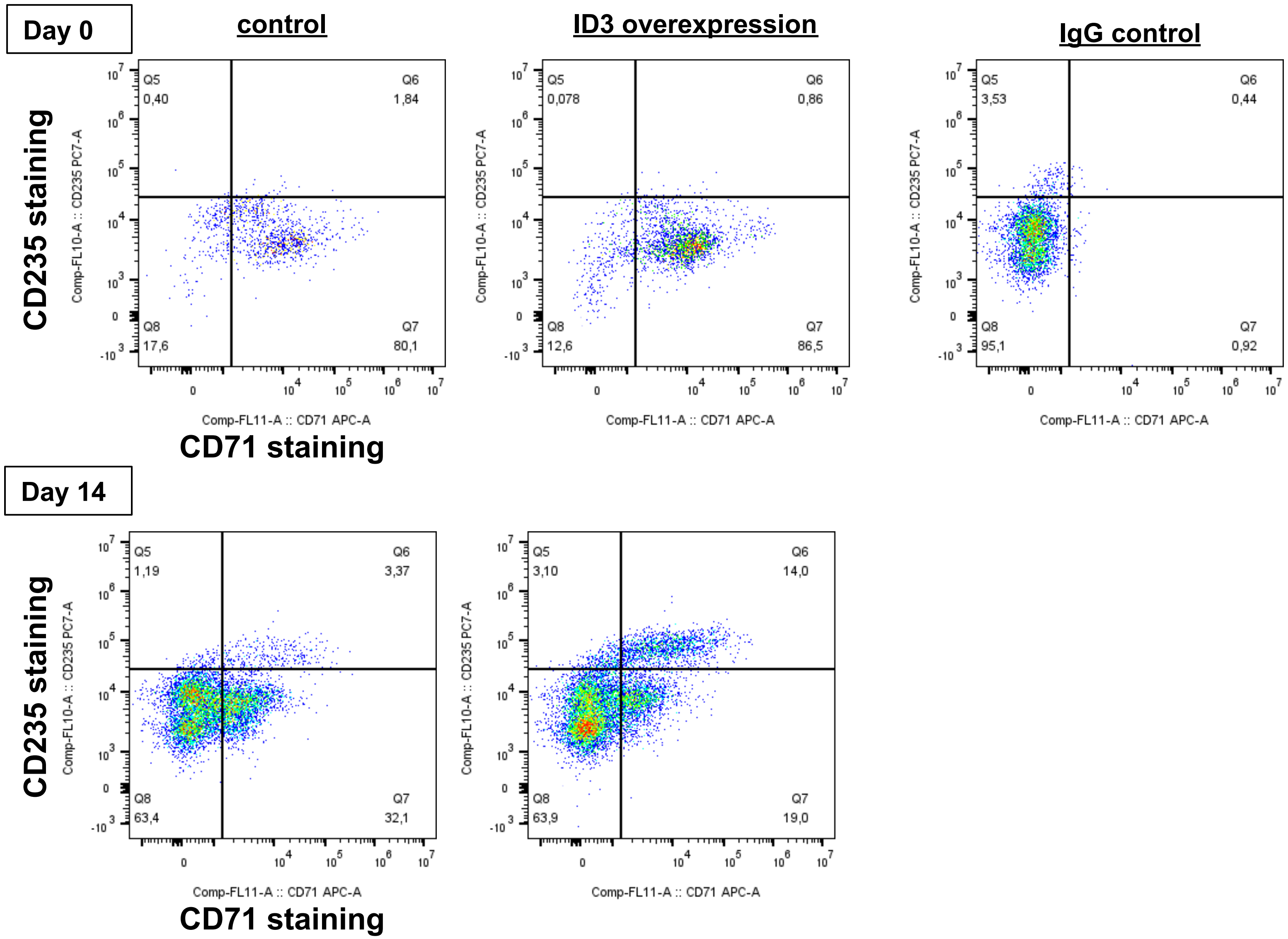

Supplementary Figure S12: ID3 overexpression leads to erythroid differentiation. (A) Differentiation of hCD34+ cells was monitored by flow cytometry. For CD34 staining IgG control, day 0 and day 14 is shown. CD34 was decreased upon differentiation to erythrocytes. CD71 was increased after 14 days of differentiation, as well as CD235. **(B)** Flow data shows GFP signal on day 0 and day 14 for control and ID3 overexpressing cells. Vector used in this experiment is LeGO IG2, which contains GFP cassette. **(C)** Flow data corresponding to bar chart. CD235 staining and CD71 staining is shown. Day 0 compared to day 14 for hCD34+ cells control and ID3 overexpressing ones.

Supplementary Table 1

shRNA		
Target	backbone	Mature Antisense Sequence
shP6#1	pInducer10-mir-RUP-PheS	TTCTTTCAGGTCTCTCTCT
shP6#2	pInducer10-mir-RUP-PheS	TGAGTATACTTTTCTAGCT
shTAL1 #1	pInducer10-mir-RUP-PheS	CAATTCGGAACATAGACCA
shTAL1 #2	pInducer10-mir-RUP-PheS	ATCACTCTACTATCATTGT
shcontrol	pInducer10-mir-RUP-PheS	CTTACTCTCGCCCAAGCGAGA

Supplementary Table 2

shRNA #2		
Target	backbone	shRNA Sequence 5´-→ 3´
shTAL1 #1	SEW/WPRE	ACCTCGACAAGAAGCTCAGCAAGAATTCAAGAGATTCTTGCTGAGCTTCTTGTCTTTTT
shTAL1 #2	SEW/WPRE	ACCTCAAAGTTGTGCGGCGTATCTTCTCAAGAGGAAGATACGCCGCACAACTTTTT
shP6#1	SEW/WPRE	ACCTCGGCATTCTGAGCATCTTCTGTTCAAGAGACAGAAGATGCTCAGAATGCCTT
shP6#2	SEW/WPRE	ACCTCGGAGGAGAAGACCAAAGACTTTCAAGAGAAGTCTTTGGTCTTCTCCTCCTT

Supplementary Table 3

Flow cytometry antibodies:

Antibody	Label	Clone	Cat.No.	Supplier	Dilution	Concentration
CD235a	PE/Cy7	HI264	349112	BioLegend	1:20	100 µg/ml
CD71	APC	CY1G4	334108	BioLegend	1:640	50 µg/ml
CD34	APC-EF780	4H11	47-0349-41 /2	eBioscience	1:20	0.5 µg/test

Supplementary Table 4

Immunofluorescence antibodies and reagents:

Antibody	Host	Clone	Cat.No.	Supplier	Dilution	Concentration
TAL1	mouse	mono	04-123	Merck/Sigma	1:250	
PRMT6	rabbit	poly	720142	Invitrogen	1:200	0,5 mg/ml
Alexa 488	rabbit	poly	A32731	Invitrogen		2 mg/ml
Alexa 647	mouse	poly	A32787	Invitrogen		2 mg/ml
Phalloidin-iFluor 555			ab176756	Abcam	1:1000	
DAPI			D8417	Sigma-Aldrich	1:5000	5 mg/ml

Supplementary Table 5

real time primers

Name	Sequence 5' →3'
GYPA fw	CCCTCCAGAAGAGGAAACCGGAGA
GYPA rev	GGCACGTCTGTGTCAGGTGAGG
PRMT6 fw	TCTGGTTCCAGGTGACCTTC
PRMT6 rev	AGGTAGAGGAGCGCCTGTTT
TAL1 fw	TCGGCAGCGGGTTCTTTGGG
TAL1 rev	CCATCGCTCCCGGCTGTTGG
E47 fw	GGGACTCGGAGGCAAGAGCG
E47 rev	TCGCCTGACAGGAAGCCAGC
CD41 fw	AATGGCCCCTGCTGTCGTGC
CD41 rev	TGCACGGCCAGCTCTGCTTC
GAPDH fw	TCTTTTGCGTCGCCAGCCGAGC
GAPDH rev	TGACCAGGCGCCCAATACGACC
ID3 fw	CAGCGCGTCATCGACTACA
ID3 rev	GTGAGCTCGGCTGTCTGGAT
CD42b fw	CTCCTGCTGCCAAGCCCCTT
CD42b rev	GTCTGGAGGCAGCGCTGTCA
CD71 fw	AGGACGCGCTAGTGTTCTTC
CD71 rev	CCAGGCTGAACCGGGTATATG
PRDM1 fw	CCCGGAGAGCTGACAATGAT
PRDM1 rev	TGGGACATTCTTTGGGCAGA
ACVRL1 fw	CACCGAGTTCGTCAACCACT
ACVRL1 rev	AGGTTGGGTGGCCTCCAG

Supplementary Table 6

ChIP primers

Name	Sequence 5' →3'
ID3 prom fw	CGCCTTGTTCCCCAATTTGC
ID3 prom rev	ACCTCAGCTTCACCGCAATTA
non targeting fw	GAGTGAGCTGGTTCTGGGTC
non targeting rev	AAGTGTGCTTTTGGCTGCTG
GYPA fw	CAGGCGCTTAACAACTTGCATCA
GYPA rev	CATACATCCTGAGATCATGAGCTG

Supplementary Table 7

Primary antibodies:

Antibody	Host	Cat.No.	Supplier	Dilution	Concentration
HA.11 Clone 16B12	mouse	901502	BioLegend	1:1000	1 mg/ml
Flag	rabbit	F7425	Merck/Sigma	1:1000	0.8 mg/ml
GAPDH loading Control	mouse	MA5-15738	Invitrogen / Thermo Fisher	1:1000	1 mg/ml
TAL1 (E-4)	mouse	sc-365527	Santa Cruz	1:200	200 µg/ml
PRMT6	rabbit	720142	Invitrogen / Thermo Fisher	1:1000	0.5 mg/ml

Supplementary Table 8

Secondary antibodies:

Antibody	Host	Cat.No.	Supplier	Dilution	Concentration
anti-mouse IgG H&L (HRP)	goat	ab97040	Abcam	1:10000	0.5 mg/ml
anti-rabbit IgG H&L (HRP)	goat	ab97080	Abcam	1:10000	0.5 mg/ml
anti-mouse IgG + IgM H&L (HRP)	goat	ab47827	Abcam	1:10000	1 mg/ml
IRDye 800CW Goat anti-Mouse IgG (H + L)	goat	926-32210	Li-Cor	1:20000	1 mg/ml
IRDye 800CW Goat anti-Rabbit IgG (H + L)	goat	926-32211	Li-Cor	1:20000	1 mg/ml

Supplementary Table 9

ChIP antibodies:

Antibody	Host	Cat.No	Supplier	ChIP	Concentration
Rabbit igG Isotype Control	rabbit	02-6102	Invitrogen / Thermo Fisher	4 µg	5 mg/ml
TAL1	rabbit	PA5-30586	Invitrogen / Thermo Fisher	4 µg	1 mg/ml
PRMT6	rabbit	720142	Invitrogen / Thermo Fisher	2 µg plus	0.5 mg/ml
PRMT6 (Middle Region)	rabbit	ABIN2778658	antkörper-online.de	2 µg	0.5 mg/ml
TCF3	rabbit	PA5-78190	Invitrogen / Thermo Fisher	4 µg	1 mg/ml
Histone H3	rabbit	PA549579	Invitrogen / Thermo Fisher	2 µg	1 mg/ml
Histone H3 asymmetric di methyl R2	rabbit	ab175007	Abcam	2 µg	1 mg/ml
Histone H3 mono methyl K9	rabbit	ab176880	Abcam	2 µg	2.168 mg/ml
Histone H3 tri methyl K4	rabbit	ab8580	Abcam	2 µg	1 mg/ml
